# *Fkbp5* gene deletion: Circadian rhythm profile and brain proteomics in aged mice

**DOI:** 10.1111/acel.14314

**Published:** 2024-09-03

**Authors:** Niat T. Gebru, Jennifer Guergues, Laura A. Verdina, Jessica Wohlfahrt, Shuai Wang, Debra S. Armendariz, Marsilla Gray, David Beaulieu‐Abdelahad, Stanley M. Stevens, Danielle Gulick, Laura J. Blair

**Affiliations:** ^1^ Byrd Alzheimer's Center and Research Institute Tampa Florida USA; ^2^ Department of Molecular Medicine University of South Florida Tampa Florida USA; ^3^ Department of Molecular Biosciences University of South Florida Tampa Florida USA; ^4^ Research and Development James A. Haley Veterans Hospital Tampa Florida USA

**Keywords:** aging, FKBP51, neuroinflammation, neuropsychiatric disorders, proteomics, stress response, synaptic transmission

## Abstract

FKBP51, also known as FK506‐binding protein 51, is a molecular chaperone and scaffolding protein with significant roles in regulating hormone signaling and responding to stress. Genetic variants in *FKBP5*, which encodes FKBP51, have been implicated in a growing number of neuropsychiatric disorders, which has spurred efforts to target FKBP51 therapeutically. However, the molecular mechanisms and sub‐anatomical regions influenced by FKBP51 in these disorders are not fully understood. In this study, we aimed to examine the impact of *Fkbp5* ablation using circadian phenotyping and molecular analyses. Our findings revealed that the lack of FKBP51 did not significantly alter circadian rhythms, as detected by wheel‐running activity, but did offer protection against stress‐mediated disruptions in rhythmicity in a sex‐dependent manner. Protein changes in *Fkbp5* KO mice, as measured by histology and proteomics, revealed alterations in a brain region‐ and sex‐dependent manner. Notably, regardless of sex, aged *Fkbp5* KOs showed elevated MYCBP2, FBXO45, and SPRYD3 levels, which are associated with neuronal‐cell adhesion and synaptic integrity. Additionally, pathways such as serotonin receptor signaling and S100 family signaling were differentially regulated in *Fkbp5* KO mice. Weighted protein correlation network analysis identified protein networks linked with synaptic transmission and neuroinflammation. The information generated by this work can be used to better understand the molecular changes in the brain during aging and in the absence of *Fkbp5*, which has implications for the continued development of FKBP51‐focused therapeutics for stress‐related disorders.

## Graphical abstract

This study investigates the circadian rhythm and proteomic profile of aged FKBP51 knockout mice. The absence of FKBP51 provided protection against stress‐mediated disruptions in circadian rhythms in a sex‐dependent manner. Proteomic changes in these mice showed brain region‐ and sex‐dependent alterations, with elevated levels of proteins like MYCBP2, FBXO45, and SPRYD3 associated with neuronal‐cell adhesion and synaptic integrity.

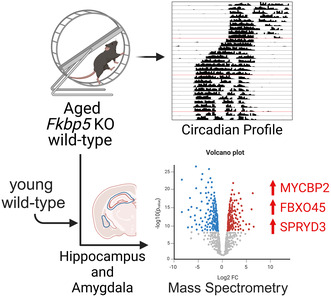

**Abbreviations**
ANOVA: analysis of variance
BMAL1: brain and muscle Arnt‐like protein‐1
CK1δ: casein kinase I isoform delta
CLOCK: circadian locomotor output cycles kaput
CORT: corticosterone
CT: circadian time
DEPs: differentially expressed proteins
DIA: data‐independent acquisition
DPBS: Dulbecco's phosphate‐buffered saline
ELISA: enzyme‐linked immunosorbent assay
FDR: false discovery rate
FKBP51: FK506‐binding protein 51
GCs: glucocorticoids
GO: gene ontology
GR: glucocorticoid receptors
HPA: hypothalamic‐pituitary‐adrenal
Hsp90: heat shock protein 90
IPA: ingenuity pathway analysis
KO: knockout
LC‐MS/MS: liquid chromatography‐tandem mass spectrometry
LFQ: label‐free quantification
MANOVA: multivariate analysis of variance
ME: module eigengene
PASEF: parallel accumulation‐serial fragmentation
PER1: period circadian protein homolog 1
PER2: period circadian protein homolog 2
PFA: paraformaldehyde
PRM: parallel reaction monitoring
PTSD: post‐traumatic stress disorder
QTOF: quadrupole time‐of‐flight
SPSS: statistical package for the Social Sciences
TIMS: trapped ion mobility spectrometry
UHPLC: ultra‐high performance liquid chromatography
WPCNA: weighted protein correlation network analysis
WT: wild‐type
ZT: zeitgeber time

## 1 INTRODUCTION

The 51 kDa FK506‐binding protein, FKBP51, is a co‐chaperone of the 90 kDa heat shock protein (Hsp90), which functions as a scaffolding protein that regulates steroid hormone signaling and stress responses (Touma et al., 2011). This ubiquitous protein demonstrates region‐specific expression patterns throughout the central nervous system (Scharf et al., 2011). FKBP51 and its encoding gene, *FKBP5*, have drawn significant scientific attention due to their links to various stress‐related disorders, including anxiety, depressive disorders, post‐traumatic stress disorder (PTSD), obesity, and suicide (Fries et al., 2017).

The role of FKBP51 in stress response regulation, particularly its influence on the hypothalamic–pituitary–adrenal (HPA) axis, has been extensively studied. FKBP51 regulates glucocorticoid receptors (GR) by reducing the sensitivity and reactivity to glucocorticoids (GCs) (Hoeijmakers et al., 2014; Touma et al., 2011). Genetic variations in the *FKBP5* gene, in combination with early life stressors, are associated with altered susceptibility to stress‐related mental health disorders (Fries et al., 2017). At least one of these variants exacerbates *FKBP5* expression following stress, implicating elevated levels of FKBP51 as a detrimental factor. Mice lacking FKBP51 (*Fkbp5* KO) have improved metabolism (Stechschulte et al., 2016), sleep profile (Albu et al., 2014), stress response dynamics (Albu et al., 2014; Hoeijmakers et al., 2014; O'Leary et al., 2011; Sabbagh et al., 2014; Touma et al., 2011), reduced neuroinflammation, and perform better in stress‐associated behavioral tasks (Hoeijmakers et al., 2014; O'Leary et al., 2011; Sabbagh et al., 2014; Touma et al., 2011). Sex‐dependent changes in *Fkbp5* KOs have been reported (van Doeselaar et al., 2023), which complement the clinical studies showing sex biases in the vulnerability to mental health disorders (McLean et al., 2011). There have been significant efforts aimed at inhibiting FKBP51 for the development of therapeutics for mental health and other disorders. A deeper understanding of the on‐target molecular and behavioral changes in the brains of aged *Fkbp5* KO mice is necessary to assess the long‐term implications of inhibiting FKBP51.

The goal of this study was to use a multi‐pronged approach to carefully dissect the impact of *Fkbp5* ablation on the brain to further validate FKBP51 as a therapeutic target. To do this, aged mice lacking FKBP51 were assessed for general homeostasis using circadian phenotyping and molecular changes using immunohistochemistry and proteomics compared to wild‐type littermates. Proteomic analysis revealed FKBP51‐regulated pathways and proteins not previously described and alterations in the aged brain, some of which were reversed by the absence of FKBP51. Weighted protein correlation network analysis (WPCNA) was carried out to further identify protein network modules co‐regulated with specific phenotypes. Overall, these findings suggest that long‐term FKBP51 ablation causes subtle, brain‐region‐specific changes, which paves the way for further investigation into its potential as a therapeutic target for stress‐related disorders.

## 2 MATERIALS AND METHODS

### 2.1 Animals

*Fkbp5* KO (*n* = 12/sex) and wild‐type (*n* = 11–12/sex) littermates were bred and aged to 17‐month‐old in‐house colony on a C57BL/6J background. The *Fkbp5* KO mice were as previously described (O'Leary et al., 2011) and are commercially available from Jackson Labs (#017989). Mice were group housed in a 12‐h light/dark cycle with ad libitum access to food and water until the start of this study. For proteomics analysis, brain tissues were collected from 3‐month‐old background‐matched wild‐type mice (*n* = 4–5/sex). All procedures were carried out according to the National Institute of Health Guide for the Care and Use of Laboratory Animals and approved by the University of South Florida Institutional Animal Care and Use Committee.

### 2.2 Assessment of circadian activity

For circadian phenotyping, mice were individually housed in chambers (Tecniplast, West Chester, PA, USA) with running wheels, wheel sensor, and ad libitum access to food and water. Wheel activity was recorded for 9 days on a 12‐h light/dark cycle (LD). LD Baseline was calculated for the final 6 days. Subsequently, mice underwent a 7‐h phase advance followed by 9 days of re‐entrainment. Phase advance was calculated as the number of days each mouse took to adjust their activity onset 7 h earlier. Next, the mice were exposed to a 24‐h dark cycle (DD) for 9 days. DD baseline free‐running period was calculated using the last 3 days. At the end of the dark cycle, mice were restrained for 10 min at CT02 and returned to their home cage for continued activity monitoring in DD (DD Stress). Welfare checks during the dark cycle utilized infrared goggles and a red light (630 nm, wavelength below the threshold for mouse perception). Data were recorded in 5‐min bins using Scurry Activity Monitor (Lafayette Instruments, Lafayette, IN, USA). The circadian period for each mouse was computed from ClockLab by calculating the slope of a regression line based on successive circadian activity onsets using the Chi‐squared periodogram (Bushell and Sokolove method). Relative amplitude (RA), interdaily stability (IS), and intraday variability (IV) were computed using nonparametric calculations based on the ratio of the average square error of hourly means from the grand mean divided by the sample variance. RA measures the difference between the 10 most active days and the five least active days divided by the summation of the two, which provides a readout for the strength and consistency of the rhythm. IS measures how well the daily activity patterns resemble one another from 1 day to the next and ranges from 0, representing inconsistent rhythm, to 1, representing high rhythm consistency. IV measures rhythm fragmentation or frequency of transitions between rest and activity within 24 h and ranges from 0, corresponding to stable rhythms, to 2, representing greater rhythm fluctuations. These are computed by the non‐parametric circadian rhythm analysis module within ClockLab. The average activity profiles were generated for LD baseline, DD baseline, and DD stress using ClockLab, which averaged the activity amplitudes for each hour in 60 min bins.

### 2.3 Tissue collection

The onset of activity was determined for each mouse from ClockLab the day before tissue harvest. Mice were euthanized by EUTHASOL overdose (Virbac, Westlake, Texas, USA) within a 2‐h window beginning at circadian time (CT) 02, which represents activity offset plus 2 h. Whole blood was collected via cardiac puncture for CORT (corticosterone) determination, followed by transcardiac saline perfusion. Brain tissue was collected; one hemibrain was flash frozen while the other was fixed using 4% paraformaldehyde (PFA) and stored in DPBS with 10 mM sodium azide until further analysis.

### 2.4 Corticosterone ELISA assay

Serum was separated from whole blood using serum separator tubes (BD, Franklin Lakes, NJ, USA) by centrifugation for 15 min at 2000 × *g* at 4°C. Serum CORT (corticosterone) was diluted 1:100 and quantified using an ELISA kit (Enzo Life Sciences, Farmingdale, NY, USA), as recommended by the manufacturer.

### 2.5 Immunohistochemistry

PFA‐fixed hemibrains were cryoprotected using sucrose gradients, and coronal sections were generated using a sliding microtome with freezing stage. Immunohistochemical staining was performed as previously described (Blair et al., 2019) using the following primary antibodies: PER1 (AB2201, MilliporeSigma, Burlington, MA, USA; 1:1000), PER2 (PA5‐100107, Invitrogen, Waltham, MA, USA; 1:1000), BMAL1 (14020S, Cell Signaling Technology, Danvers, MA, USA; 1:1000) CLOCK (PA1‐520 Invitrogen; 1:3000), and CK1δ (PA5‐32129, Invitrogen; 1:250). Stained tissues were mounted onto slides, dehydrated in alcohol gradients, and coverslipped with DPX mountant (Sigma‐Aldrich, St. Louis, MO, USA). Imaging was performed using Zeiss Axio Scan.Z1 (ZEISS Microscopy, Munich, Germany). NearCYTE (http://nearcyte.org), a pixel‐based brightfield analysis software, was used to define regions of interest (ROI) and select stain‐positive cells based on adjusted thresholds. A parametric segmentation method was used to quantify the percent area ratio of positive staining.

### 2.6 Proteomics

For proteomic analysis, 3‐month‐old wild‐type mice (*n* = 4–5/sex) were added to the existing groups (aged wild‐type; *n* = 6/sex and aged *Fkbp5* KO; *n* = 6/sex). A small portion of hippocampal tissue and amygdala was collected (~1–4 mg tissue weight) and processed using the iST sample preparation kit (PreOmics, Planegg/Martinsried, Germany) (Kulak et al., 2014) with their adapted protocol for tissue. Briefly, the tissue was submerged in 100 μL of iST LYSE buffer along with protein extraction beads (Diagenode, Denville, NJ, USA) followed by sonication (9 cycles—5 s on and 5 s off at 20% amplitude). After extraction, protein quantification was carried out using the Pierce 660 nm assay with ionic detergent combability reagent in order to normalize protein amount (~50 μg) prior to digestion with the iST digest buffer (containing trypsin/LysC). The digested sample was transferred to the iST cartridge for subsequent washing and extraction of peptides. The extracted peptides were centrifuged under vacuum at 42°C to complete dryness and then reconstituted in 0.1% formic acid in water to achieve a final concentration of 2 μg/μL for the peptide stock solution.

Samples were diluted to achieve 200 ng on‐column injections followed by separation on a nanoElute (Bruker, Billerica, MA, USA) nanoflow ultra‐high performance liquid chromatography (UHPLC) system coupled to LC–MS/MS analysis on a trapped ion mobility spectrometry (TIMS)‐QTOF instrument (timsTOF Pro, Bruker). A column oven heated to 50°C was utilized with a CaptiveSpray ion source using the Aurora Ultimate CSI UHPLC reversed‐phase C18 column (25 cm × 75 μm i.d., 1.7 μm C18, IonOpticks). Mobile phases A (0.1% formic acid in water) and B (0.1% formic acid in acetonitrile) were utilized in a method where the total runtime was 120 min. Specifically, peptide elution occurred during a 90‐min gradient of 2%–25% B, and the remaining time was used to ramp up to 37%–80% B to clean the column and prepare for the next blank and subsequent sample runs. DIA‐PASEF scan mode was implemented to operate the timsTOF Pro within an ion mobility range of 0.7–1.40 1/K0 [V·s/cm^2^] spanning 250–1425 m/z, resulting in an estimated 1.48 s cycle time. Additional settings included a mass width of 25 Da, one mobility window, no mass or mobility overlap, and collision energy and DIA‐PASEF windows were 20 eV for a base of 0.60 1/K0 [V·s/cm^2^] and 59 eV for a base of 1.60 1/K0 [V·s/cm^2^]. Calibration for ion mobility and m/z were performed linearly using three ions at 622, 922, and 1222 m/z (Agilent). For PRM validation, the timsTOF Pro was operated in PRM‐PASEF mode using the same LC method as the DIA analysis to cover the targets selected based on pathway‐specific and biological relevance of differentially expressed proteins. Peptide concentration normalization was achieved through injection volume adjustment using peptide assay results of the protein digests. PRM‐PASEF parameters included an ion mobility range and m/z range of 0.7–1.2 1/K0 [V·s/cm^2^] and m/z 378–931, respectively, as well as collision energy setting of were 20 eV for a base of 0.70 1/K0 [V·s/cm^2^] and 59 eV for a base of 1.40 1/K0 [V·s/cm^2^].

DIA data were analyzed in DIA‐NN (v.1.8.1) in library‐free mode using an in silico library generated from the Uniprot *Mus musculus* database (UP000005640, 55,315 entries). The match‐between‐runs (MBR) feature was implemented within the label‐free quantification (LFQ) approach using an FDR cutoff of 1% with the following settings selected: single‐pass mode neural network classifier, genes as the protein inference, robust LC (high precision) as the quantification strategy, cross‐run normalization that is RT‐dependent, and smart profiling library generation. Skyline v.23.1.0.268 was used to analyze PRM data compared against the DIA library generated from DIA‐NN. Transition settings included quantitation based on top 3 fragment b‐ or y‐type fragment ions, library ion match tolerance of 0.1 m/z, MS/MS filtering for PRM acquisition with 30,000 resolving power and high‐selectivity extraction enabled, retention time filtering using scans within 5 min of MS/MS IDs, and ion mobility library matching using 30 resolving power.

### 2.7 Weighted protein Co‐expression network analysis (WPCNA)

Weighted protein Co‐expression network analysis was performed on circadian behavior, hippocampal immunohistochemistry, and proteomic data using WGCNA package in R (v.1.72–5) (Langfelder & Horvath, 2008). This involved transforming the signed topological overlap matrix (TOM) to determine connectivity in the adjacent matrix and categorizing proteins into modules based on TOM‐based dissimilarity measurements. Key module identification parameters included soft‐threshold power = 7, merge Cut Height = 0.25, and minimal module size = 20, resulting in 20 modules containing hub proteins and their co‐expressed counterparts. Proteins from modules most correlated with traits of interest were selected for Gene Ontology (GO) pathway analyses.

### 2.8 Statistical analysis

SPSS (v.29; Chicago, IL, USA) was used to analyze behavior and immunohistochemistry data. GraphPad Prism 10.2.0 (GraphPad Software, San Diego, CA, USA) was used to generate graphs. Circadian measurement variables (period, amplitude, relative amplitude, interdaily variability, and interdaily stability) were computed using ClockLab analysis software version 6.1 (Actimetrics, Wilmette, IL, USA).

For immunohistochemistry, group outliers were evaluated, and genotype and sex interactions were identified using multivariate analysis, followed by univariate analysis. Tukey's post hoc analysis detected significant differences. The group mean area ratio was used for SPSS analysis, and graphical representation was done using GraphPad Prism 10.2.0 (GraphPad Software, San Diego, CA, USA).

For proteomics, statistical testing and bioinformatic analysis were performed as previously described (Guergues et al., 2020) in Perseus (v.1.6.15.0) with minor changes to accommodate for DIA‐NN results for each individual experiment and subsequent search (i.e., female amygdala, female hippocampus, male amygdala, male hippocampus). Specifically, the contaminants from each DIA‐NN pg. matrix output file were removed, and the remaining protein groups were uploaded to Perseus. The label‐free quantification (LFQ) intensity values were log_2_ transformed and annotated into separate aged *Fkbp5* KO, aged wild type, or young wild‐type groups before being filtered 60% in at least one group (i.e., 60% valid LFQ values were present in at least one group). Missing values were replaced through the imputation function (replace missing values from normal distribution option within Perseus) with a width of 0.3 and a downshift of 1.8 to fit the lower abundance of the Gaussian curve (Deeb et al., 2012). This filtering approach was implemented to increase sensitivity of differentially expressed protein detection of low abundance proteins but also to capture FKBP51 depletion in subsequent bioinformatic analyses (measured as significant downregulation based on imputed LFQ values that were originally missing in *Fkbp5* KO groups). Welch's *t*‐test (*p*‐value cutoff of <0.05) was used to compare aged KO to aged wild‐type to determine the changes of the *Fkbp5* KO on aged mice and then to the aged wild‐type to the young wild‐type to determine the effect of aging without genetic manipulation. GO annotation was then applied, and the resulting list was exported. To increase confidence for subsequent validation (i.e., false discovery rate (FDR) control), an additional *z*‐score cutoff of >1 was also implemented in addition to Welch's t‐test significance cutoff (Ramus et al., 2016). Proteins that met both Welch's *t*‐test and *z*‐score cutoffs were uploaded to Ingenuity Pathway Analysis (IPA) for bioinformatic analysis. IPA‐generated enrichment of canonical pathways was filtered for *p* < 0.05 (Fisher's Exact Test) and *z*‐score cutoff >1.5. For PRM statistical analysis, total area for each experimental group and biological replicate of each peptide target was extracted from Skyline and utilized to determine statistical significance using unpaired *t*‐test (*p* < 0.05).

## 3 RESULTS

### 3.1 *Fkbp5* KO males resist stress‐induced changes in circadian period

**FIGURE 1:**
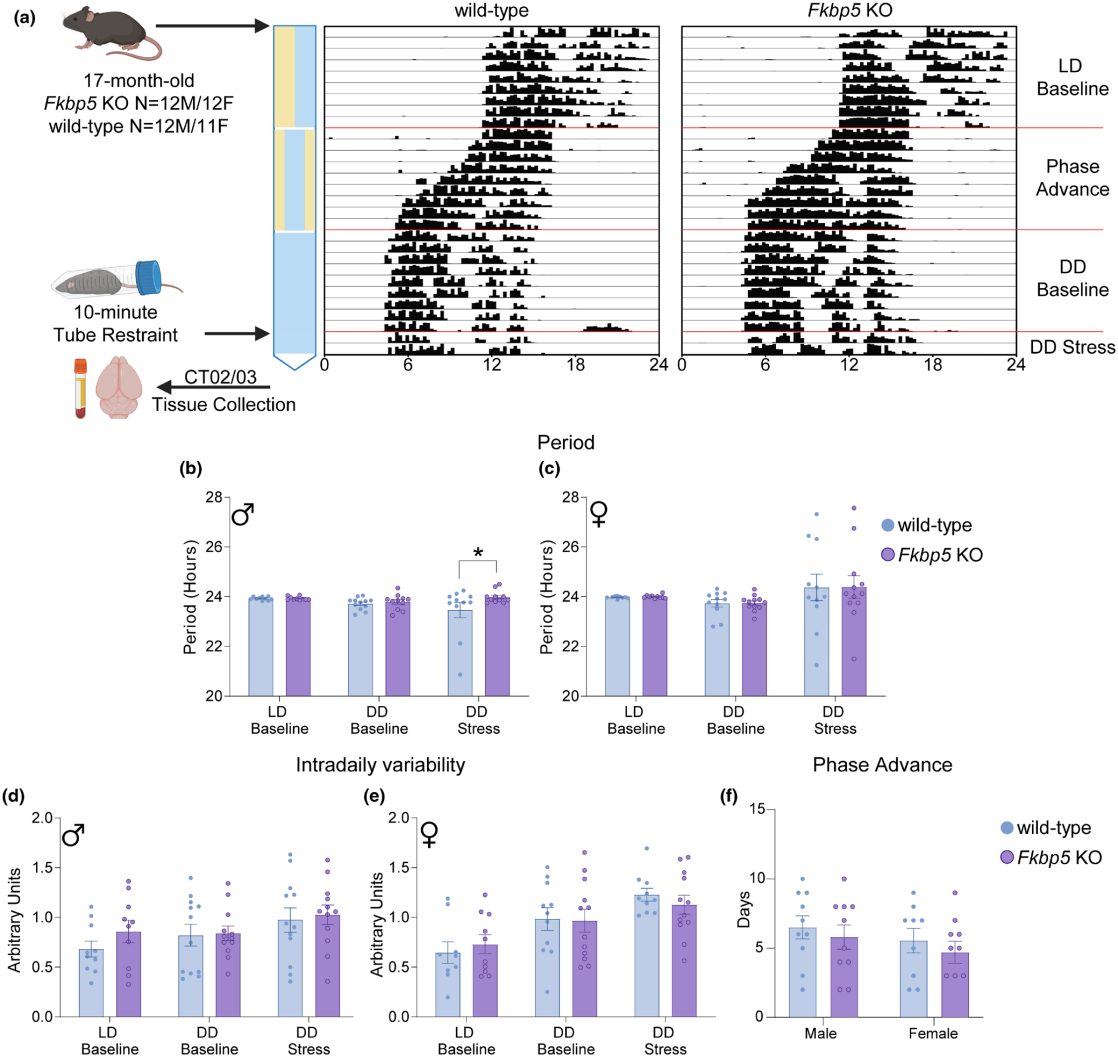
Male *Fkbp5* KO resist stress‐induced changes in circadian period length. (a) Aged 17‐month‐old male and female *Fkbp5* KO and wild‐type mice were housed in individual circadian phenotyping cages. Wheel running activity was measured as a proxy of circadian rhythmicity during 12‐h light/dark (LD baseline), 7‐h phase advance, and 24‐h darkness (DD baseline). Mice were subjected to a 10‐min tube restraint 48 h before the end of DD. Representative actogram of wheel‐running data from one mouse displaying activity onset throughout the experimental period. Length of circadian period in LD and DD at baseline and DD stress in (b) males and (c) females. Intradaily‐variability (IV) in LD and DD at baseline and DD stress in (d) males and (e) females. (f) Number of days taken for re‐entrainment to 7‐h phase advance by wild‐type and *Fkbp5* KO male and female mice. Data were analyzed by SPSS MANOVA followed by ANOVA with Tukey post hoc test. *Fkbp5* KO (*n* = 12/sex) and wild type (*n* = 11–12/sex). Results represented as mean ± SEM. Statistical significance is indicated by *p < 0.05.

Voluntary wheel running activity was assessed in aged *Fkbp5* KO and wild‐type littermates over the course of 30 days to examine circadian rhythmicity, which is a highly sensitive measure of physiology that is often disrupted in neuropsychiatric disorders (Walker 2nd et al., 2020). Wheel activity was recorded during periods of 12‐h light/dark cycle (LD baseline), 7‐h phase advance, or 24‐h dark cycle (DD baseline). To assess the effects of stress on the circadian rhythm, mice were subjected to 10‐min tube restraint stress at the end of DD Baseline (DD Stress) at CT 0 (Figure 1a). No differences between the genotypes were found in the circadian period in LD baseline and DD baseline conditions (Figure 1b,c), indicating proper operation of the circadian clock. However, after exposure to an acute stress, male wild‐type mice demonstrated a shortened period, whereas the *Fkbp5* KOs resisted this change (Figure 1c), suggesting that the absence of *Fkbp5* may mitigate the effects of stress on the circadian response, at least in male mice.

Next, circadian rest‐activity measurement variables were evaluated. Intradaily variability (IV) and interdaily stability (IS), which are markers of rhythm fragmentation within one circadian day and the stability of the rhythm across different days, respectively, showed no effect of sex or genotype (Figure 1d,e; Figure S1A,B). Relative amplitude (RA), a measure of the robustness of the rest‐activity rhythm, was reduced in female *Fkbp5* KOs during LD baseline compared to wild‐type females and male *Fkbp5* KOs (*p* < 0.001) (Figure S1C,D; Table S1). Overall, during the LD baseline period, the activity profile was similar between both genotypes during subjective night but during the early hours of the active period, wild‐type mice showed significantly higher amplitude ZT13, ZT14, ZT15, and ZT24 (*p* < 0.05) (Figure S1E–G; Table S1). Activity profiles between DD and DD stress were similar between the two genotypes.

Phase advance, a measure of the number of days taken to adjust to new light cues, showed no differences by genotypes or sex (Figure 1f). Repeated measure ANOVA was used to test for stress effects on the endogenous rhythm (DD baseline vs. DD stress). There was a significant main effect of stress on amplitude (*F*_(1,40)_ = 7.842, *p* < 0.01) and IV (*F*_(1,42)_ = 13.559, *p* < 0.001) but not on period, relative amplitude, or interdaily stability (Table S1).

### 3.2 BMAL1 levels are elevated *in Fkbp5* KO

**FIGURE 2:**
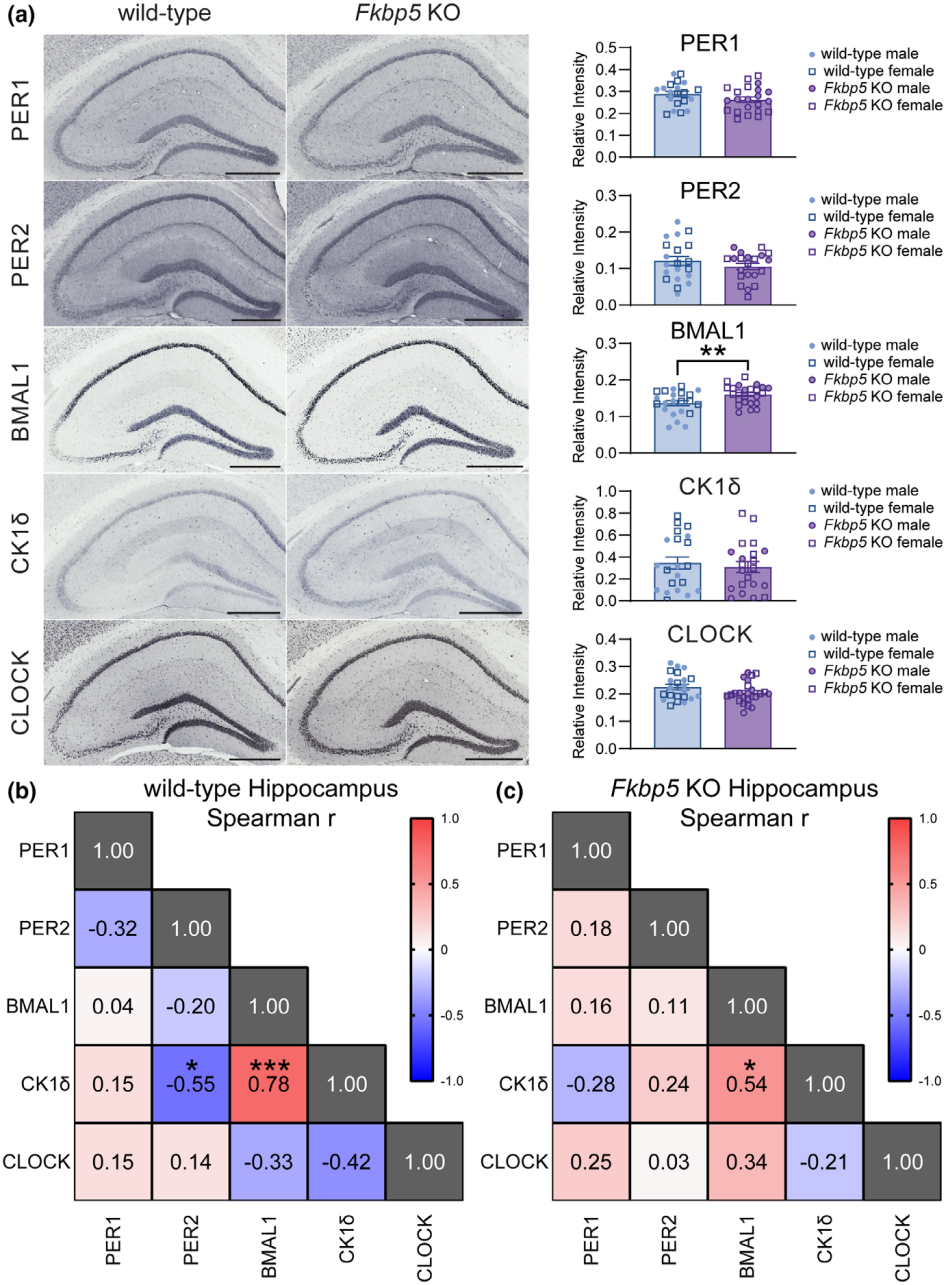
BMAL1 are elevated in the hippocampus of *Fkbp5* KOs. (a) Representative images and relative intensity quantitation from hippocampal tissues of wild‐type and *Fkbp5* KO mice (*n* = 23–24/genotype) stained with PER1, PER2, BMAL1, CK1δ, or CLOCK. Data were analyzed by two‐way ANOVA. Spearman rank correlation matrix of core clock proteins as determined by immunohistochemistry in the hippocampus of aged (b) wild‐type and (c) *Fkbp5* KO mice. Red colors represent negative correlation, blue colors represent positive correlation and white colors represent no linear correlations. Results represented as mean ± SEM. Statistical significance is indicated by *p < 0.05, **p < 0.01, ***p < 0.001.

To maintain molecular consistency, tissue collection was performed within a 2‐h window of CT02. Male and female *Fkbp5* KO mice exhibited significantly reduced basal corticosterone levels (Genotype *F*_(1,40)_ = 32.87, *p* < 0.0001) compared to wild‐type mice (Figure S2), consistent with prior reports (Albu et al., 2014; Hoeijmakers et al., 2014; Sabbagh et al., 2014). To explore molecular changes in clock protein levels within brain regions involved in stress regulation, brain tissues from the hippocampus and amygdala were stained for core clock proteins, Period circadian protein homolog 1 (PER1), PER2, Brain and muscle Arnt‐like protein‐1 (BMAL1), and circadian locomotor output cycles kaput (CLOCK) as well as casein kinase I isoform delta (CKIδ), which is a critical regulator of PER1 (Etchegaray et al., 2009). While most proteins remained unchanged by genotype in both regions, a significant upregulation of BMAL1 was observed in the hippocampus of *Fkbp5* KO mice as determined by univariate analysis (Genotype: *F*_(1,44)_ = 9.710, *p* < 0.01) (Figure 2a; Table S2). In the amygdala, PER1 levels in male *Fkbp5* KOs were increased compared to female *Fkbp5* KOs, as revealed by Tukey post hoc (*p* < 0.01) (Figure S3A,B; Table S3). No significant interactions between genotype and sex were found in either brain region (Tables S2 and S3). Since there is a delicate interplay between the levels of circadian proteins, a Spearman's rank‐order correlation was used to assess the relationship among clock proteins. In the hippocampus, wild‐type mice showed a negative CK1δ:PER2 correlation (Figure 2b), while a positive correlation was seen in the amygdala (Figure S3C). A positive correlation between BMAL1 and CK1δ was measured in the hippocampus (Figure 2b,c) and amygdala (Figure S3C,D). However, this was not an FKBP51‐mediated effect, since it was found in both genotypes.

### 3.3 Proteomic profile of aged *Fkbp5* KO and wild‐type mice

**FIGURE 3:**
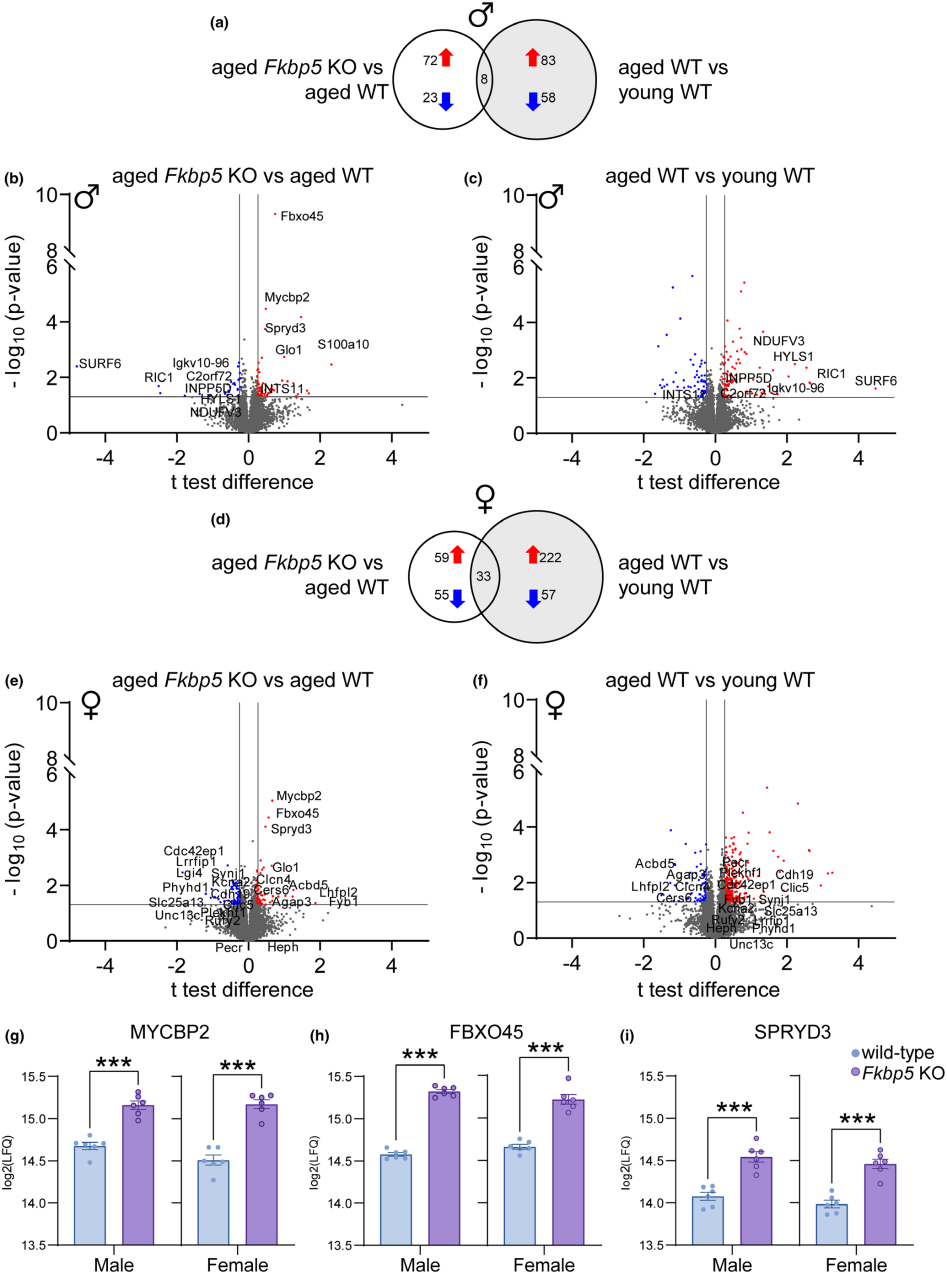
Deep proteomic analysis of hippocampus in relation to *Fkbp5* KO, aging, and sex. Venn diagram showing total number of significantly up or downregulated proteins as well as overlapping between the aged *Fkbp5* KO vERSUs aged wild type and aged wild type versus young wild type in (a) males and (d) females. Volcano plot of proteome in aged *Fkbp5* KO versus aged wild type in the hippocampus of (b) males and (e) females. Volcano plot of hippocampal proteome in aged wild type versus young wild type of (c) males and (f) females. Volcano plot x‐axis represents Welch's *t*‐test difference highlighting both significantly upregulated (red) and downregulated (blue) proteins. Y‐axis represents the −log_10_ of the corresponding *p*‐values, indicating the statistical significance associated with each observed alteration. The grey threshold lines delineate predefined criteria for Welch's *t* test difference corresponding to *z*‐score of >1 (vertical lines) and significance of *p* < 0.05 (horizontal line). (g–i) Relative quantitation of ubiquitin pathway‐associated proteins (MYCBP2, FBXO45, and SPRYD3) obtained from the deep proteomic analysis of hippocampus. Results represented as mean ± SEM. Statistical significance is indicated by ***p < 0.001.

To dissect the molecular underpinnings of FKBP51 in the aged brain, proteomic analysis of the hippocampus and amygdala was performed in *Fkbp5* KO and wild‐type mice (*n* = 5–6/genotype/sex) at 18 months of age. To determine if any of the resilient phenotypes reported in *Fkbp5* KO mice (Touma et al., 2011) are due to the maintenance of proteins in a similar state as young wild‐type mice, 3‐month‐old (*n* = 4–5/sex) wild‐type mice were also evaluated. A total of >8000 unique proteins were quantified per run with high quantitative precision (median CV = 12.6%) across the measured proteome for each group (Figure S4) and then filtered for statistically significant differentially expressed proteins (DEPs, Tables S4–S11). Overall, the effects of *Fkbp5* KO on global protein expression changes in the hippocampus and amygdala were subtle, as demonstrated by the limited number of DEPs and related magnitude of fold changes. In the hippocampus, aged *Fkbp5* KO males had 103 DEPs compared to aged wild‐type counterparts (Figure 3a,b), which showed 149 DEPs compared to young wild‐type mice (Figure 3a,c). Notably, only eight proteins overlapped between these male comparison sets (Figure 3a). Aged female *Fkbp5* KO mice displayed 147 DEPs compared to their wild‐type counterparts (Figure 3d,e), which showed 312 DEPs relative to young wild‐type mice (Figure 3d,f). Interestingly, only 33 proteins overlapped between these two female datasets (Figure 3d). In the amygdala, aged *Fkbp5* KO males had 84 DEPs compared to aged wild‐type controls (Figure 5a,b), which showed 239 DEPs compared to young wild‐type mice (Figure 5a,c). Ten proteins overlapped between these male comparison sets (Figure 5a). Aged female *Fkbp5* KO mice displayed 68 DEPs compared to their wild‐type counterparts (Figure 5d,e), which showed 157 DEPs relative to young wild‐type mice (Figure 5d,f). There were eight overlapping proteins in the amygdala between these two female data sets (Figure 5d).

**FIGURE 4:**
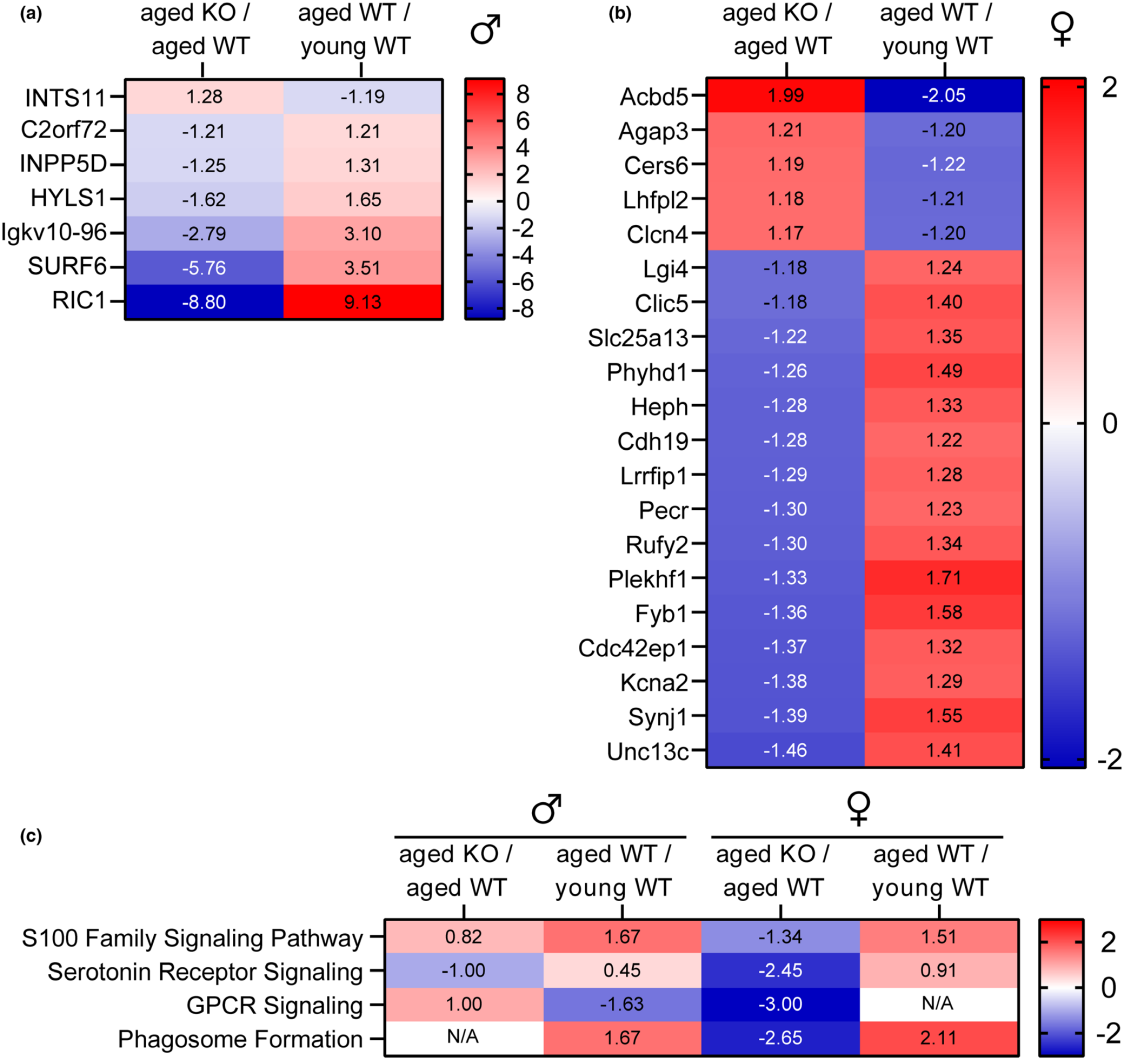
List of proteins demonstrating altered expression patterns during aging yet displaying a reversed expression profile upon *Fkbp5* KO. List of differentially expressed proteins in the hippocampus of (a) males and (b) females that overlapped between the *Fkbp5* KO versus aged wild type and aged wild type versus young wild type and corresponding fold change. Red colors represent upregulation and blue colors represent downregulation. (c) IPA analysis based on activation *z*‐score from differentially expressed proteins of *Fkbp5* KO male and female mice reveal pathways related to extracellular signaling, neuroinflammation, phagocytosis and hormone signaling (Welch's *t*‐test *p* < 0.05 and |*z*‐score|>1.5).

To investigate the potential role of preserving proteins in a young‐like state contributing to resilience in *Fkbp5* KO mice, DEPs were filtered to identify those that show contrasting expression patterns between age (aged WT/young WT) and *Fkbp5* KO (aged KO/aged WT). DEPs that meet these criteria were considered to represent proteins in the *Fkbp5* KOs that are expressed similarly to young wild‐type mice, potentially underpinning the observed resilience phenotype. Interestingly, in male *Fkbp5* KO mice, seven hippocampal proteins and nine amygdalar proteins exhibited expression patterns similar to young wild‐type mice (Figure 4a; Figure S6A). The picture was even more striking in females, with 20 proteins in the hippocampus and three proteins in the amygdala showing expression patterns mimicking young wild‐type mice (Figure 4b; Figure S6B).

Pathway‐specific proteins within the regional overlaps were unique to each sex. DIA‐based quantitation of specific DEPs in the hippocampus that were consistently changed in the *Fkbp5* KOs is shown in Figure 3. The ubiquitin‐associated Myc‐binding protein 2 (MYCBP2), F‐box/SPRY domain‐containing protein 45 (FBXO45), and SPRY Domain‐Containing Protein 3 (SPRYD3) were among the most robust and reproducible DEPs in *Fkbp5* KOs regardless of sex, showing significant upregulation in both the hippocampus and amygdala (Figure 3b,e; Figure S5B,E). Log_2_LFQ values are shown in Figure 3g–i. In order to enhance quantitative precision and validate the relative abundance of select proteins in the hippocampus, parallel reaction monitoring (PRM) was performed (Figure S7A–F). This independent quantitative validation of key target proteins provided a secondary validation of the increased MYCBP2, FBXO45, and SPRYD3 levels in the hippocampus of both male and female *Fkbp5* KO mice. Additional markers related to endocytosis that were changed in *Fkbp5* KO males (ARHGAP21, CSF1R, S1000A10, and RUFY2) or females (CSF1R, TBC1D24) were also measured and demonstrated significant (*p* ≤ 0.05) or trending (*p* ≤ 0.08) changes.

To explore the functional relevance of the DEPs identified, IPA was used to determine enriched pathways due to lack of *Fkbp5* (aged KO/aged WT) or aging (aged wild‐type/young WT) in male and female mice. Specifically, a comparison analysis was performed with the top canonical pathways based on activation *z*‐score, with at least one of the comparison groups showing *p* < 0.05 for enrichment significance. This allowed the identification of trends in pathway activity changes related to aging and FKBP51 ablation (Figure 4c; Table S12). S100 family signaling was predicted to be activated with age in both males (*z* = 1.67) and females (*z* = 1.51) while trending towards inhibition with *Fkbp5* KO males (*z* = 0.82) and females (Figure S8). Similarly, serotonin receptor signaling was trending towards predicted activation with age in males (*z* = 0.45) and females (*z* = 0.91) while showing a trend toward inhibition in the *Fkbp5* KO males (*z* = −1) and females (*z* = −2.45). On the other hand, G protein‐coupled receptor (GPCR) signaling was predicted to be inhibited with aging (*z* = −1.63) in males, but *Fkbp5* KOs show an opposite trend towards activation (*z* = 1.0). Phagosome formation was predicted to be activated with age in both males (*z* = 1.67) and females (*z* = 2.11) but inhibited with *Fkbp5* KO only in females (Figure 4c). In terms of enriched pathways related to functional outcomes, endocytosis was identified as activated with aging in both male and female groups yet showed predicted inhibition in both the male and female *Fkbp5* KO compared to wild‐type mice, selected targets shown in Figure S7G–L. In the amygdala, no specific pathways were generated by IPA for *Fkbp5* KOs (activation *z*‐score cutoff of 1.5 and *p* < 0.05).

### 3.4 *Fkbp5* KO exhibit distinct protein expression correlation patterns through WPCNA

**FIGURE 5:**
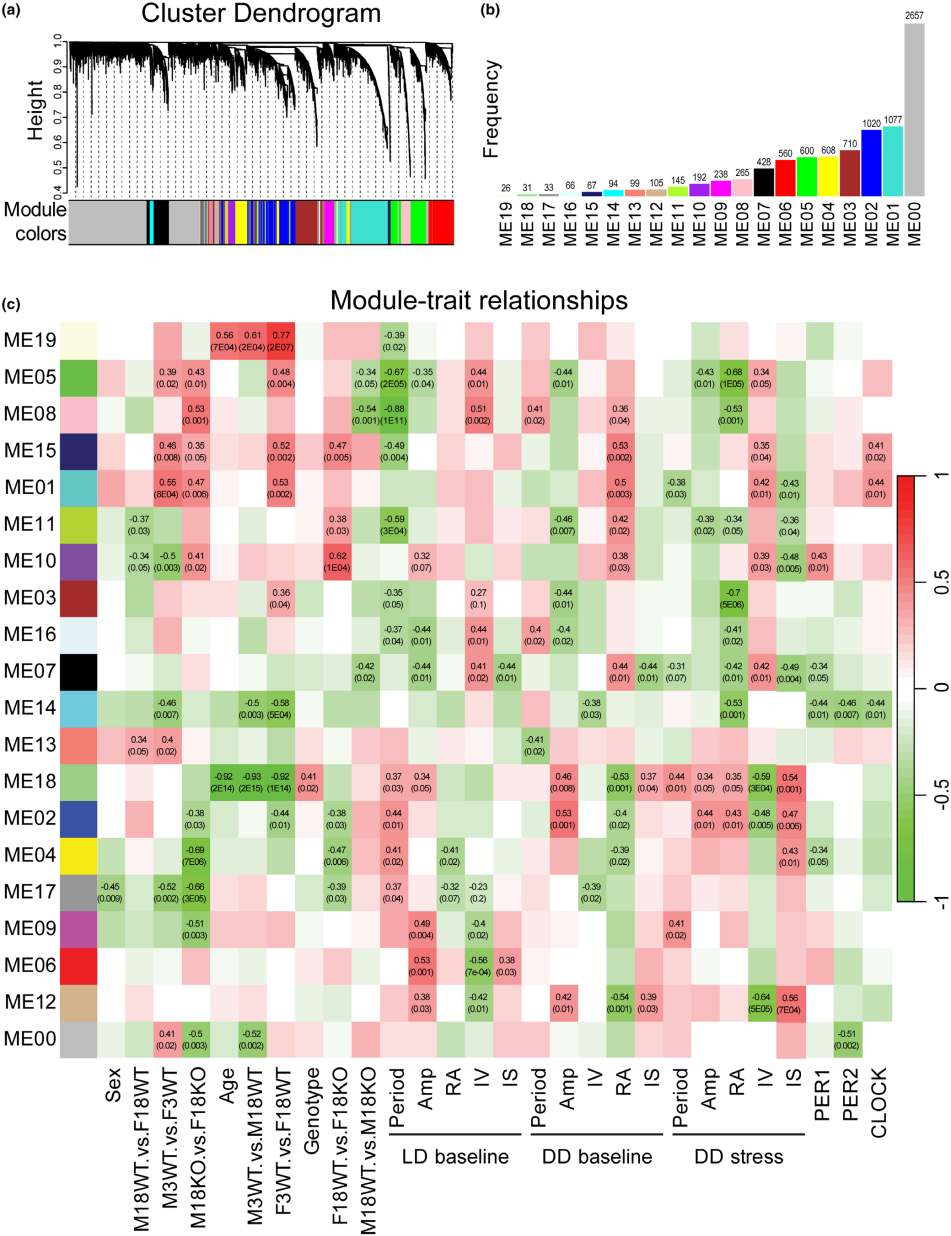
Identification of genotype‐ and sex‐specific protein networks. (a) Cluster dendrograms of all genes, with dissimilarity based on topological overlap, were assigned to various module numbers. Height = One minus Pearson correlation (1 − *r*). (b) Numbers of proteins in each module plotted as a bar plot. (c) Heatmap of module‐trait relationship. Each row and column correspond to a module eigengene or a clinical trait. Each cell contains the Pearson correlation coefficient and corresponding *p*‐value. The red color represents a positive correlation, while the green color represents a negative correlation.

WPCNA was performed to identify protein expression correlation patterns between the circadian behavior, histology (hippocampus), and proteomics (hippocampus) datasets (Langfelder & Horvath, 2008). This allowed the identification of clusters of highly correlated proteins, summarizing these clusters using the module eigengene and relating modules to external sample traits (circadian behavior and immunohistochemistry). Hierarchical clustering and dynamic tree‐cutting algorithm using WPCNA package revealed 20 module eigengene (ME0 to ME19) of co‐expressed proteins (Figure 5a,b). The number of proteins in each module ranged from 26 in ME19 to 1077 in ME01, excluding module ME00, which represented 2540 unassigned proteins (Table S13). Correlation of each variable: genotype, age, sex, and circadian measure variable at LD baseline, DD baseline, and DD stress as well as staining results (PER1, PER2, BMAL1, CK1δ, and CLOCK), with the modules was evaluated (Figure 5c).

**FIGURE 6:**
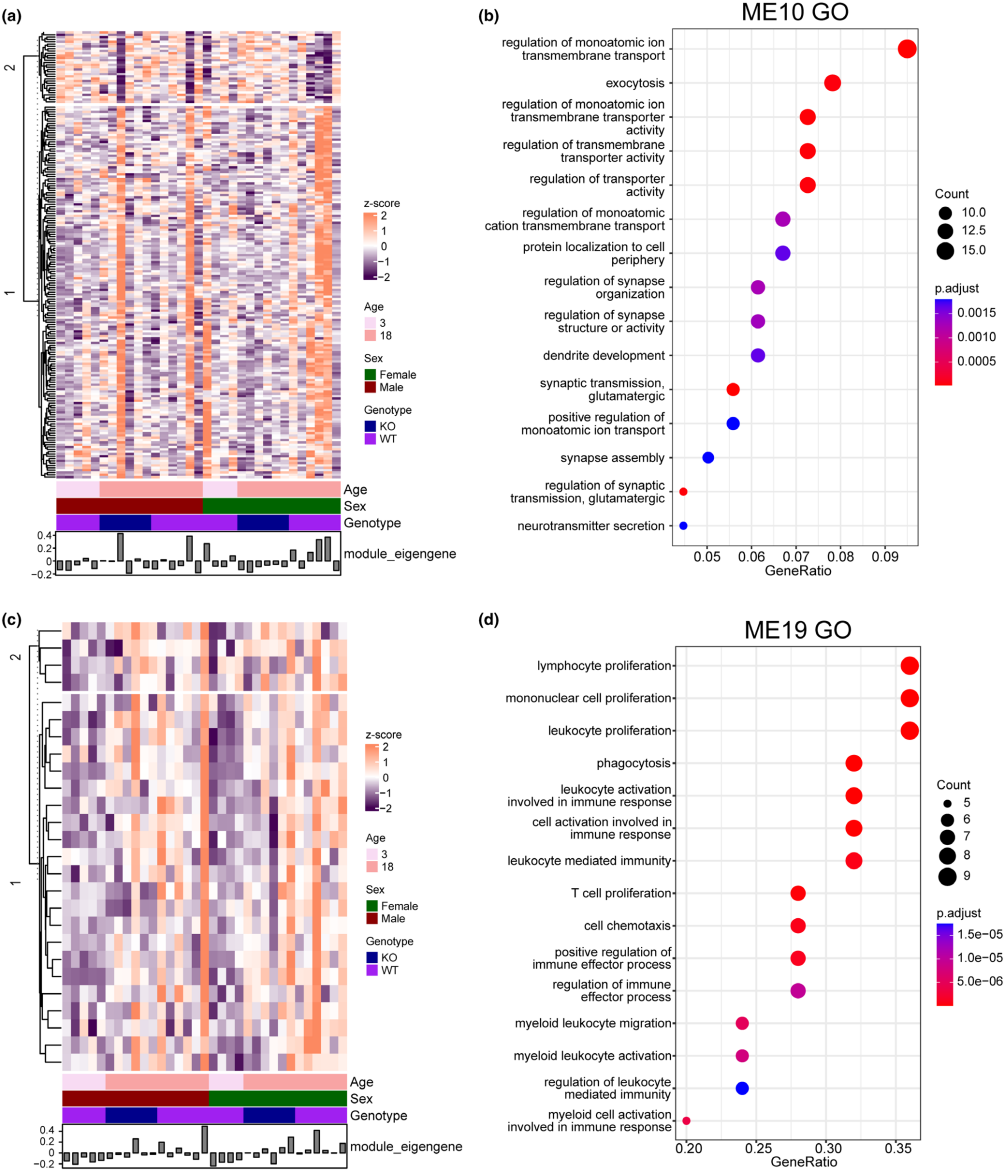
*Fkbp5* KO highly correlates with modules associated with synaptic health and immune response. (a) Heat map of protein expression profile in module ME10: Each row and column correspond to a protein or a sample. The color from violet to orange means the *z*‐score of a specific protein among all the samples in this study. The module eigengene is considered a representative of the gene expression profiles in a module. (b) Top 15 GO biological processes of module ME10 with the most significant adjusted *p*‐value are plotted in order of protein ratio. The color of the dots represents the adjusted *p*‐value of the GO term, and the size of the dots represents gene counts corresponding to the GO term. (c) Heat map of protein expression profile in module ME19: Each row and column correspond to a protein or a sample. The color from violet to orange means the *z*‐score of a specific protein among all the samples in this study. The module eigengene is considered a representative of the gene expression profiles in a module. (d) Top 15 GO biological processes of module ME19 with the most significantly adjusted *p*‐value are plotted in order of protein ratio. The color of the dots represents the adjusted *p*‐value of the GO term, and the size of the dots represents gene counts corresponding to the GO term.

A significant correlation (*p* < 0.05) was found between these variables and modules for sex (ME01 and ME17), genotype (ME18), and age (ME18 and ME19) (Figure 5c). In addition to these correlations, ME10 was negatively correlated with young and aged wild‐type male mice, but this trend was reversed in aged *Fkbp5* KO males (Figure 5c). GO terms within ME10 were primarily associated with the transmembrane transport of ions and synaptic organization (Figure 6a,b; Table S14). Similar correlations were identified in module ME01 between young wild‐type and aged *Fkbp5* KO mice but not in aged wild‐type mice (Figure S9A,B; Table S15). This further supports commonalities between aged mice lacking FKBP51 and young wild‐type mice. Another interesting observation was the correlation of age with module M19, which is primarily associated with activation and proliferation of immune response cells, indicating age‐related changes in immune cell function and composition (Figure 6c,d; Table S16).

Notably, the fragmentation of the circadian rhythm, IV, exhibited a significant positive correlation against modules ME07 and ME12 across all three light phases, which was inverse to IS, a measure of rhythm stability (Figure 5c). Top GO terms in module ME12 are mainly associated with cellular metabolism and response to stress (Figure S9C,D; Table S17) while ME07 is associated with RNA processing (Figure S10B,C; Table S18). Module ME14 exhibited a negative correlation with clock proteins, PER1, PER2, and CLOCK (Figure 5c) and was associated with GO terms related to ribosomal assembly, RNA processing, translation processes at synaptic terminals (Figure S10C,D; Table S19).

## 4 DISCUSSION

This study provides additional support towards validating FKBP51 as a therapeutic target. The lack of FKBP51 did not alter overall rhythmicity and protected from this stress‐induced disruption in aged male mice. The impact of FKBP51 ablation on specific proteins was modest, as evidenced by the small number of DEPs with a fold change greater than 2 and varied depending on brain region and sex associated. Multiple DEPs were associated with ubiquitination and endocytosis. Notably, the most affected cellular processes were associated with neurotransmission and neuroinflammation.

Interestingly, despite the predominantly sex‐ and region‐specific nature of differentially expressed proteins in *Fkbp5* knockout mice, a small group of proteins (MYCBP2, FBXO45, and SPRYD3) was consistently upregulated in both sexes and brain regions. MYCBP2, a large E3 ubiquitin ligase, supports the maintenance of cytoskeletal dynamics in axonal development and synaptic integrity (Grill et al., 2016). MYCBP2 and FBXO45 interact with cadherin as a complex to promote neuronal differentiation (Na et al., 2020). Recent data suggest SPRYD3 may also be included in this cadherin complex with SPRYD3 mediating binding between FBXO45 and the SPRY motif of N‐cadherins to initiate neuronal migration and maturation (Na et al., 2020). The MYCBP2:FBXO45 complex has also been shown to stabilize Ephrin receptor B2 (EphB2) signaling (Chang et al., 2024). The E3 ligase property of MYCBP2 is not necessary for EphB2 binding, and, counterintuitively, loss of MYCBP2 drives ubiquitin‐mediated EphB2 lysosomal degradation. Neuropsin, a stress‐inducible serine protease, facilitates the extracellular cleavage of EphB2 (S. Chang et al., 2016), which then binds N‐methyl‐D‐aspartate (NMDA) receptors to activate downstream signals involved in synaptic plasticity, the induction of *Fkbp5*, and anxiety‐like behavior (Attwood et al., 2011). We hypothesize that a MYCBP2:EphB2 complex may function in a similar manner. Loss of MYCBP2 function (either knockout or variants of the gene) is characterized by lower synaptic density and abnormal synaptic morphology (AlAbdi et al., 2023). Also detrimental, FBXO45 knockout causes abnormal innervation of the diaphragm and impaired synapse formation at neuromuscular junctions (Saiga et al., 2009). Thus, elevated levels of MYCBP2, FBXO45, and SPRYD3 in aged *Fkbp5* KOs may contribute to the resilience phenotype by maintaining neuronal‐cell adhesion and synaptic integrity via N‐cadherin and EphB2 signaling.

Unsurprisingly, Glyoxalase 1 (*Glo1*) levels were also consistently upregulated in *Fkbp5* KO mice. This has been reported in prior studies and transcriptomics analysis of the hippocampus and medial prefrontal cortex in the *Fkbp5* KOs (Kollmannsberger et al., 2013; Kwon et al., 2019). It was determined that this is not a result of FKBP51 manipulation but instead due to a gene duplication event that occurred when making the model originally (Kollmannsberger et al., 2013).

S100 family signaling in *Fkbp5* KOs was found to be maintained in a state more similar to young wild‐type mice compared to aged‐matched controls. This is not the first time that S100 family proteins have been linked to FKBP51. S100A6 can mediate protein phosphatase 5C (PPP5C) and FKBP51 interaction, which regulates endothelial barrier function (Haldar et al., 2020). Here, we report that *Fkbp5* ablation in male mice may result in aged‐associated rescue of inflammatory response in the hippocampus through the S100 signaling pathway. FKBP51 has been established as a direct interactor and negative regulator of nuclear factor kappa B (NFκB) (Erlejman et al., 2014). Interestingly, elevated levels of S100A10 (2.3‐fold) in male *Fkbp5* KOs, result in the predicted activation of NFκB and downstream signaling involving cell migration, survival, and inflammatory response. On the contrary, endogenous FKBP51 levels in aged wild‐type mice result in elevated S100A4 (1.9‐fold) in males and S100B (1.6‐fold) in females, which predicts inhibition of NFκB. This is in line with prior studies that demonstrated that S100A10 can interact with the serotonin receptor, 5‐hydroxytryptamine receptor 1b (HTR1B), to influence the onset of depressive states (Svenningsson et al., 2006). *S100a10* KO mice show depressive‐like behavior, which is rescued by restoring activity of S100A10 (Alexander et al., 2010). Increased levels of S100A10 in male *Fkbp5* KOs may be mediating the protection from depressive‐like behaviors that has been reported by our group and others in this model (Hoeijmakers et al., 2014; Kwon et al., 2019; O'Leary et al., 2011; Sabbagh et al., 2014). Serotonin receptor signaling, which is involved in mood regulation, sleep, and appetite, showed an age‐related activation in wild‐type mice that was blunted in *Fkbp5* KOs. This is in line with prior murine studies reporting increased levels of serotonin receptors, HTR2C and HTR3A, with age (Lee et al., 2000). High serotonin levels are linked to the activation of inflammatory mediators, including interleukin‐6 (IL‐6), which has been suggested to cause depression through modulation of the HPA axis (Ting et al., 2020). Elevated IL‐6 has also been reported in cases of chronic stress, while IL‐6 KO mice, like *Fkbp5* KOs, are protected from depressive‐like behavior (Monje et al., 2011). Thus, the reduced activation of the serotonin receptor signaling pathway may contribute to the anti‐depressive phenotype in *Fkbp5* KO mice.

From the WPCNA analysis, modules 10 and 19 were perhaps with the most interesting results in the context of synaptic health maintenance, M10, which had a significant correlation with *Fkbp5* KO, and M19, which was heavily associated with the immune system with a significant correlation with aging. M10 showed a negative correlation with both the young and aged wild‐type groups but a positive correlation with the aged *Fkbp5* KO group, suggesting that a subset of the protein network was altered solely by lack of *Fkbp5* regardless of age. Enrichment analysis in M10 resulted in GO terms associated with synaptic transmission, axonogenesis, ion transport, and cell projection organization. Prior work has described the role of FKBP51 in regulating GR transcriptional activity (Hoeijmakers et al., 2014; Touma et al., 2011). FKBP51 has been suggested to affect chaperone‐mediated AMPA receptor recycling and contribute to altered synaptic dynamics (Blair et al., 2019). Furthermore, FKBP51's involvement in signaling pathways related to interaction with Hsp90, NF‐κB activation, and AKT signaling is critical for neuronal survival, synaptic plasticity, and axiogenesis (Kastle et al., 2018; Wang, 2011). Additionally, the function of FKBP51 in regulating the GR signaling pathway may intersect with the GO terms related to ion transport and synaptic transmission, as glucocorticoid signaling has also been linked to these processes (Fries et al., 2017).

Module M19 showed a positive correlation with aging regardless of sex. GO terms associated with this module show enrichment of pathways related to immune cell activation, proliferation, migration, and effector functions, suggesting alterations in neuroinflammation. This could indicate a compensatory response to age‐related declines in immune function, aiming to maintain immune surveillance and combat infections or possibly diseases. Notably, microglia‐expressed proteins, ionized calcium‐binding adaptor molecule 1 (Iba1 or Aif1), and cathepsin S, as well as glial fibrillary acidic protein (GFAP) in astrocytes were enriched in this module. Changes in these proteins have been well established with age as well as neurodegeneration (Wendt et al., 2008; Xie et al., 2013). Other enriched pathways in this module include myeloid cell activation, leukocyte migration, and phagocytosis, suggesting an increased activation and recruitment of innate immune cells potentially due to “inflammaging,” a hallmark of aging (Satyam & Bairy, 2022). Overall, this module highlights the interplay between immune activation and dysregulation in the aging process.

Consistent correlations were observed between intradaily variability and interdaily stability in modules ME07 and ME12, which suggests the presence of independent networks of non‐overlapping proteins influencing these phenotypes. This aligns with the concept of phenotypes, like vulnerability, being influenced by multiple interdependent networks (Nakamura et al., 2019).

Genetic disruption of core circadian clock genes has been shown to impair hippocampal‐dependent memory (Wardlaw et al., 2014). Specifically, BMAL1 has been linked to dendritic spine formation and neuroprotection in rodent models (Ikeno & Nelson, 2015). Studies in non‐human primates have also linked BMAL1 ablation with elevated cortisol levels and depression‐related behavior phenotypes (Qiu et al., 2019). Thus, increased BMAL1 in *Fkbp5* KOs by immunohistochemistry may contribute to the anti‐depressive phenotype. However, there was no change in BMAL1 levels by proteomics, which is possibly due to the different detection methods used by these approaches. PER1, CLOCK, and CK1δ consistently showed no change in the hippocampus by genotype or sex between these methods. The elevated levels of PER1 found in the amygdala of male *Fkbp5* KO mice compared to female *Fkbp5* KOs may contribute to some of the sex differences found in this model. In male rats, acute stress decreased PER1 levels in the amygdala (Al‐Safadi et al., 2014). Therefore, the higher levels in male *Fkbp5* KOs may influence resilience in this model. This effect is not observed in the hippocampus, which may suggest that GR‐mediated PER1 activation is greater in the amygdala in the absence of *Fkbp5*, particularly in males. Comparison to the proteomic dataset was not possible since PER1 was not detected among the ~8000 proteins measured in the amygdala. BMAL1, CLOCK, and CK1δ levels were consistently unchanged in the amygdala by genotype or sex. While this study provides valuable insights into clock protein dynamics in stress‐related brain regions at a single time point, future investigations should expand upon these findings to elucidate the temporal dynamics and interactions that govern these correlations in greater detail.

In conclusion, this study links FKBP51 and MYCBP2, FBXO45, and SPRYD3. This work also highlights the importance of sex as a biological variable since most of the changes identified were sex‐dependent. WPCNA revealed the connections between the phenotypic and molecular outcomes, providing evidence for specific protein networks that correlate with the resilience found in aged FKBP51 KO.

## AUTHOR CONTRIBUTIONS

Conceptualization: LJB Methodology: NTG, JG, SMS, DG, LJB; Data curation: NTG, JG, SMS, LJB; Formal analysis: NTG, JG, SMS, DG, LJB; Investigation: NTG, JG, LAV, JW, SW, DSA, MG, DBA; Writing – Original Draft Preparation: NTG, JG, SMS, LJB; Writing – Review & Editing: NTG, JG, SMS, DG, LJB; Visualization: NTG, JG, SW, SMS, LJB; Supervision: SMS, DG, LJB; Project Administration: LJB; Funding Acquisition: LJB.

## FUNDING INFORMATION

This work was supported by the Department of Veterans Affairs, Veterans Health Administration, Office of Research and Development; Award Number 1I01 BX004626. The views expressed in this article are those of the authors and do not necessarily reflect the position or policy of the Department of Veterans Affairs or the United States government. This work was also supported by 2R01 NS073899, which is co‐funded by the National Institute of Neurological Disorders and Stroke and the National Institute of Aging. The content is solely the responsibility of the authors and does not necessarily represent the official views of the National Institutes of Health. This work was also supported in part by the Alzheimer's Association AARG‐22‐974,562.

## CONFLICT OF INTEREST STATEMENT

The authors declare that they have no conflict of interest.

## Supporting information


Figures S1–S10.

Tables S1–S19.

## Data Availability

The mass spectrometric data have been deposited to the ProteomeXchange Consortium via the PRIDE (Perez‐Riverol et al., 2022) partner repository with the dataset identifier PXD049439.
