# Supplementary material for: Fkbp5 gene deletion: Circadian rhythm profile and brain proteomics in aged mice
Source: Aging Cell. 2024 Sep 3;23(12):e14314. doi: 10.1111/acel.14314 (PMC11634734; doi:10.1111/acel.14314)
Supplement: Supplementary file 1 — Figures S1–S10. [file ACEL-23-e14314-s001.docx]

**Supporting Information**

***Fkbp5* Gene Deletion: Circadian Rhythm Profile and Brain Proteomics in Aged Mice**

Niat T. Gebru^1,2^, Jennifer Guergues^3^, Laura A. Verdina^1,2^, Jessica Wohlfahrt^3^, Shuai Wang^1,2^, Debra S. Armendariz^1,2^, Marsilla Gray^1,2^, David Beaulieu-Abdelahad^1,2^, Stanley M. Stevens Jr^3^, Danielle Gulick^1,2^, Laura J. Blair^1,2,4^

^1^Byrd Alzheimer's Center and Research Institute, Tampa, Florida, USA

^2^Department of Molecular Medicine, University of South Florida, Tampa, Florida, USA

^3^Department of Molecular Biosciences, University of South Florida, Tampa, Florida, USA

^4^Research and Development, James A. Haley Veterans Hospital, Tampa, FL, USA

Correspondence:

Laura J. Blair

4001 E. Fletcher Ave.

Tampa, Florida 33613

813-396-0639

laurablair@usf.edu

**Supplementary Figures**

**
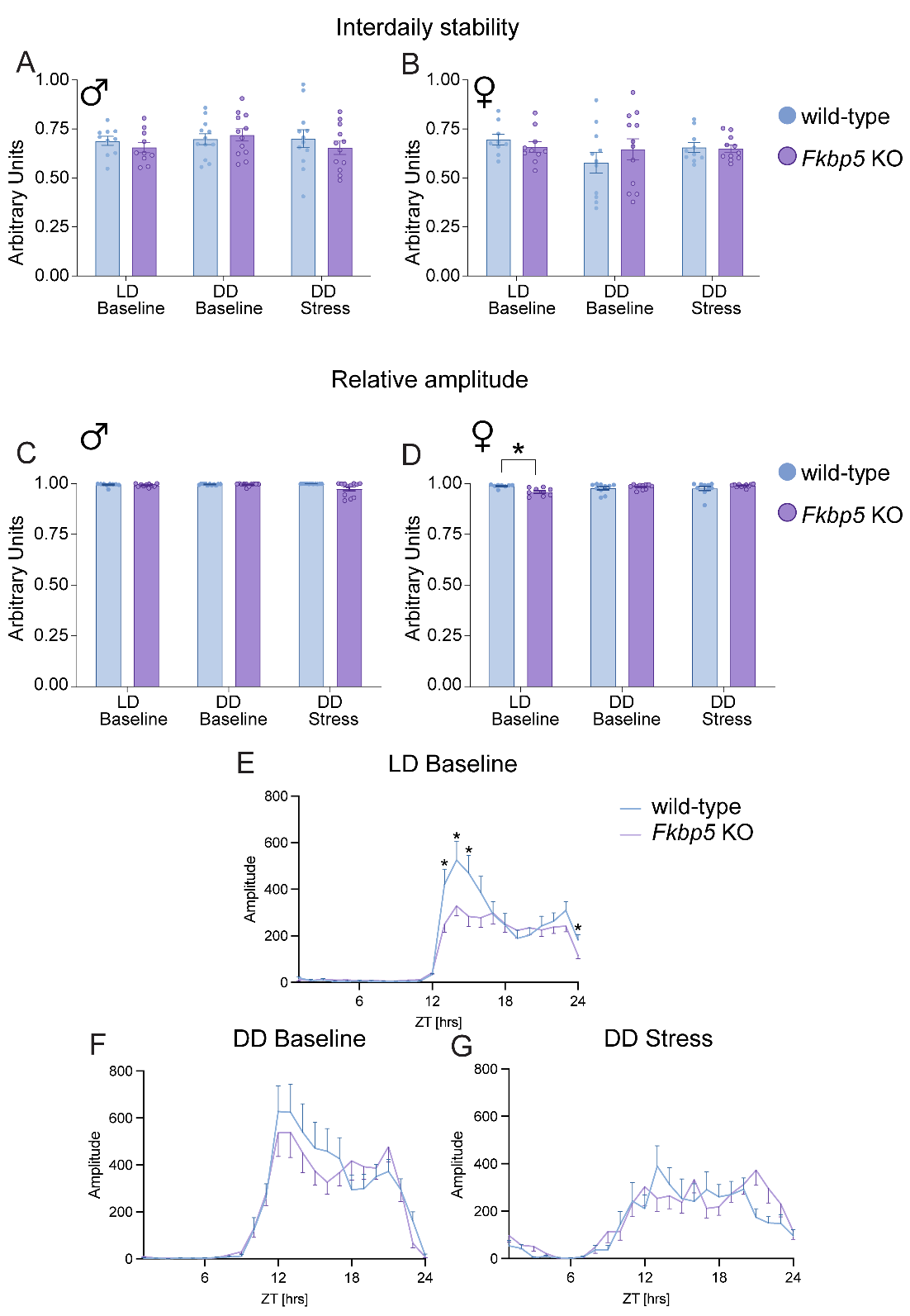
**

**Figure S1:**  **Rhythm interdaily stability and relative amplitude are similar between *Fkbp5* KO and wild-type mice.** Wheel-running activity was used to determine non-parametric circadian variables – interdaily stability (IS) in **A** males and **B** females (n =10-12/sex) and relative amplitude (RA) of LD baseline, DD baseline and DD stress in **C** males and **D** females (n =10-12/sex). Circadian rhythm activity profile of *Fkbp5* KO and wild-type mice during **E** LD baseline, **F** DD baseline, and **G** DD stress. Data were analy­­­zed by SPSS MANOVA followed by ANOVA with Tukey post hoc test. Results represented as mean ± SEM. Statistical significance is indicated by *p < 0.05.


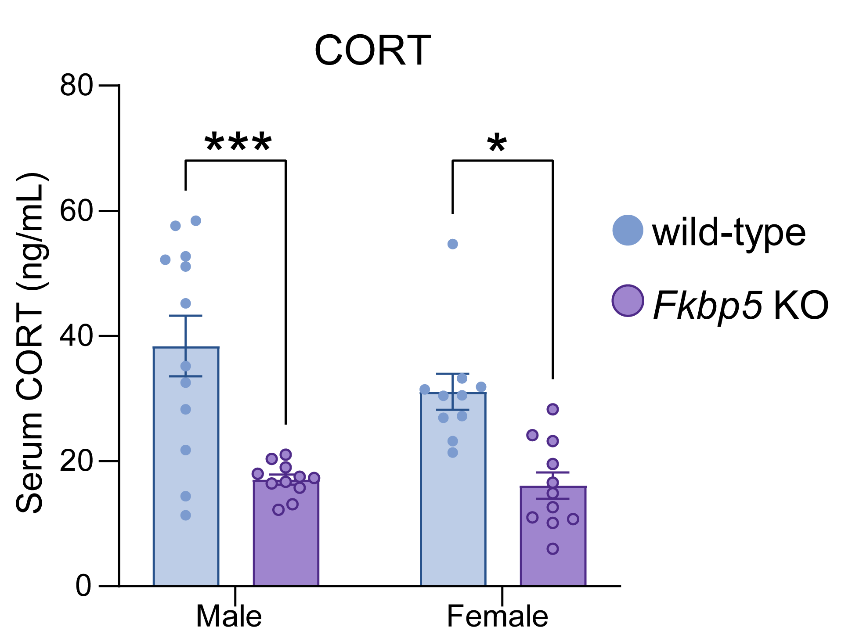


**Figure S2: *Fkbp5* KOs have reduced corticosterone levels.** Total serum CORT (corticosterone) levels (ng/mL) in wild-type (n = 12 males and 9 females) and *Fkbp5* KO (n = 11 males and 11 females) mice as determined by ELISA. Data were analyzed by Two-way ANOVA with Tukey post hoc test. Results represented as mean ± SEM. Statistical significance is indicated by *p < 0.05, ***p < 0.001.


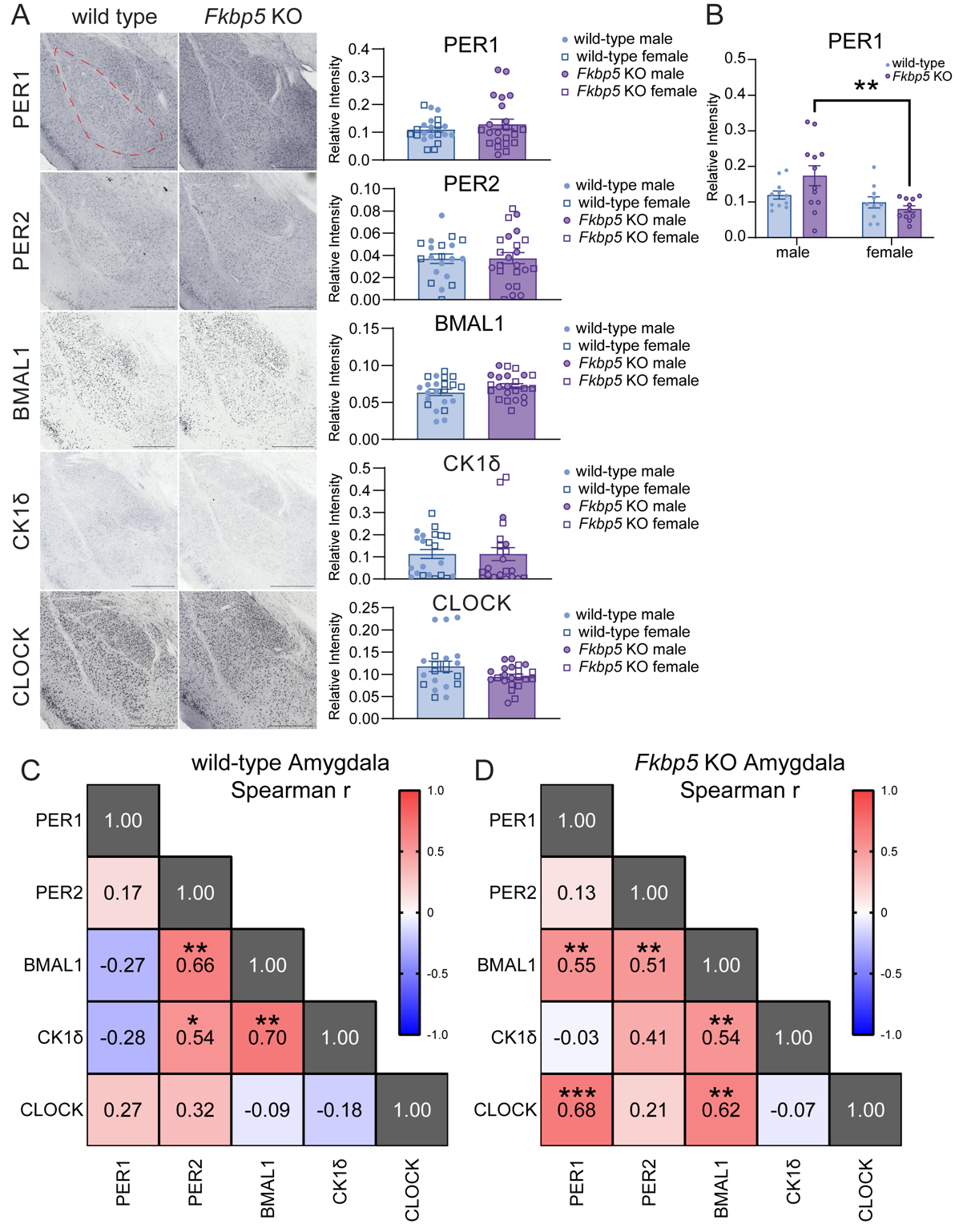


**Figure S3: Sex-specific alteration in PER1 protein in the amygdala.** **A** Representative figures and relative intensity quantitation from amygdala tissues of wild-type and *Fkbp5* KO mice (n = 23-24/genotype) stained with PER1, PER2, BMAL1, CK1δ, or CLOCK. **B** PER1 quantification separated by sex. Data were analy­­­zed by multivariate analysis, followed by univariate analysis and Tukey posthoc where applicable. Spearman rank correlation matrix of core clock proteins as determined by immunohistochemistry in the hippocampus of aged **C** wild-type and **D** *Fkbp5* KO mice. Red colors represent strong negative correlation, blue colors represent strong positive correlation, and white colors represent no linear correlations. Statistical significance is indicated by *p < 0.05, **p < 0.01, ***p < 0.001.


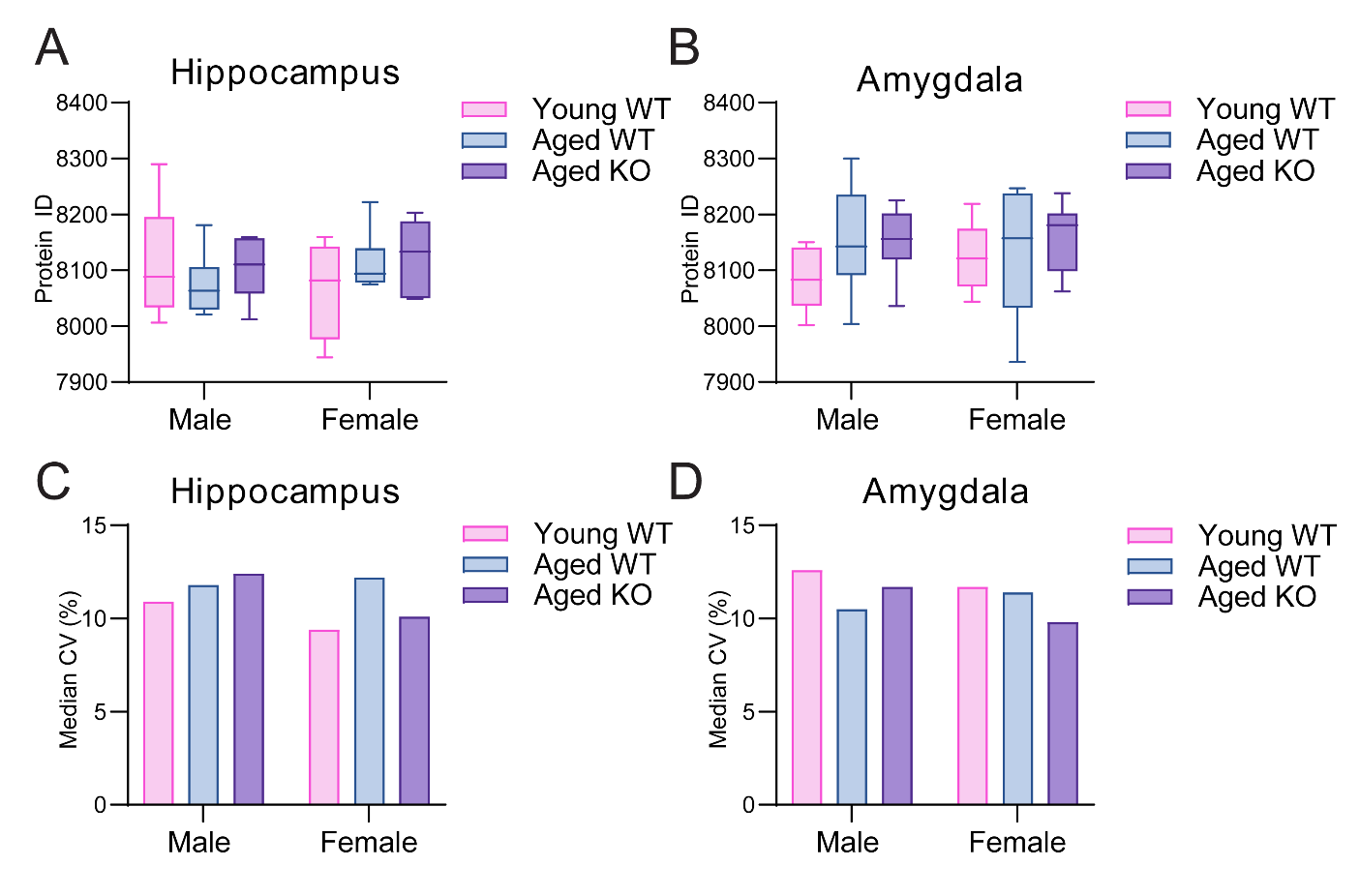


**Figure S4:**  **Proteomics coverage and related quantitative precision.** Total number of unique proteins detected in the **A** hippocampus and **B** amygdala (separated based on sex and genotype) as well as related median coefficient of variation (%CV) of LFQ values measured across the entire proteome of **C** hippocampus and **D** amygdala.


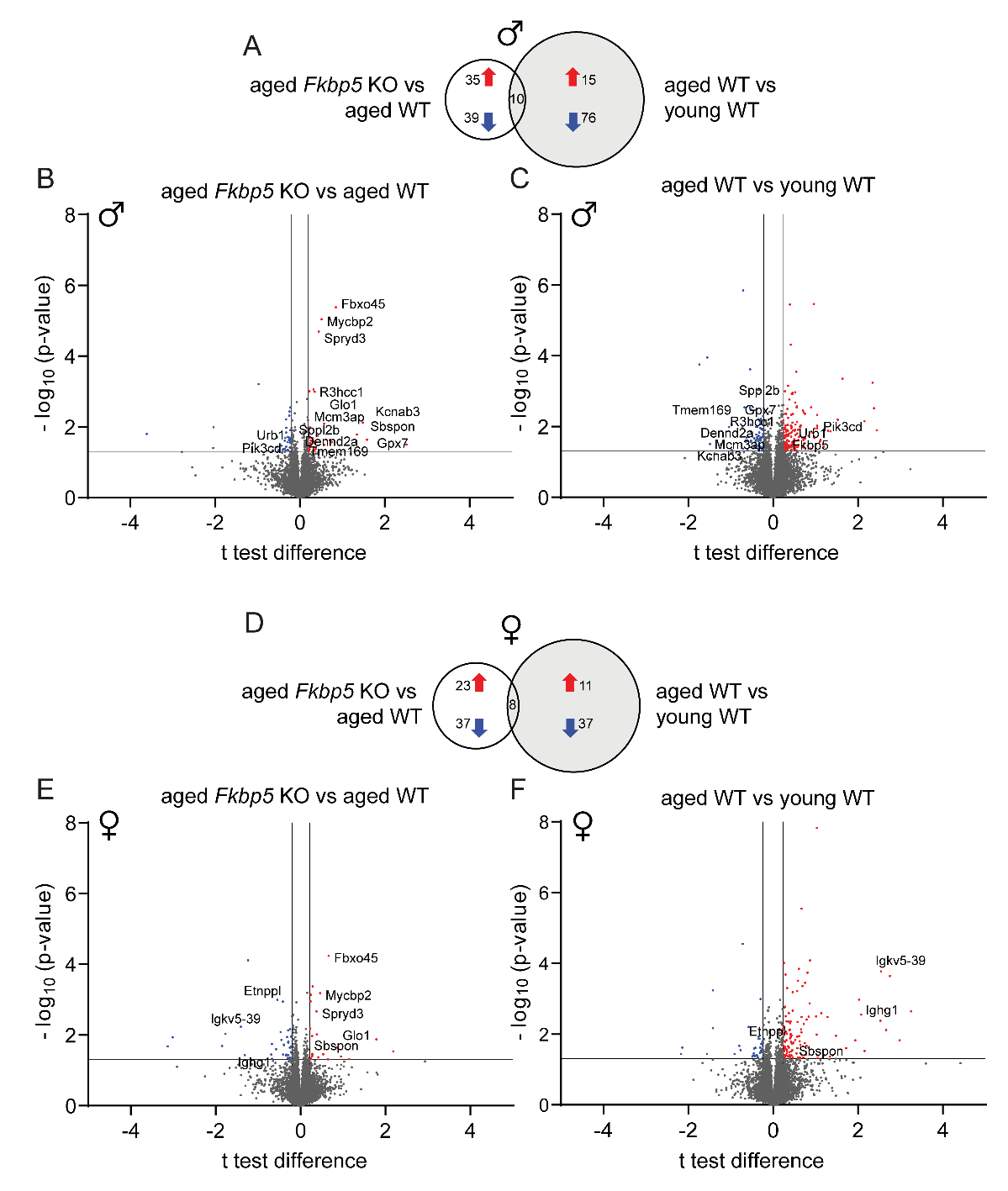


**Figure S5: Deep proteomic analysis of amygdala in relation to *Fkbp5* KO, aging, and sex. A** Venn diagram showing total number of significantly up- or downregulated proteins as well as overlapping between aged *Fkbp5* KO vs aged wild-type and aged wild-type vs young wild-type in males. Volcano plot of amygdala proteome in aged *Fkbp5* KO vs aged wild-type in the amygdala of **B** male *Fkbp5* KO vs aged wild-type and **C** male aged wild-type vs young wild-type. **D** Venn diagram showing total number of significantly up- or downregulated proteins as well as overlapping between aged *Fkbp5* KO vs aged wild-type and aged wild-type vs young wild-type in females. Volcano plot of amygdala proteome in aged wild-type vs young wild-type in the amygdala of **E** female *Fkbp5* KO vs aged wild-type and **F** female aged wild-type vs young wild-type. Volcano plot X-axis represents Welch’s t test difference highlighting both significantly upregulated (red) and downregulated (blue) proteins. Y-axis represents the -log_10_ of the corresponding p-values, indicating the statistical significance associated with each observed alteration. The grey threshold lines delineate predefined criteria for Welch’s t test difference corresponding to z-score of > 1 (vertical lines) and significance of p < 0.05 (horizontal line).


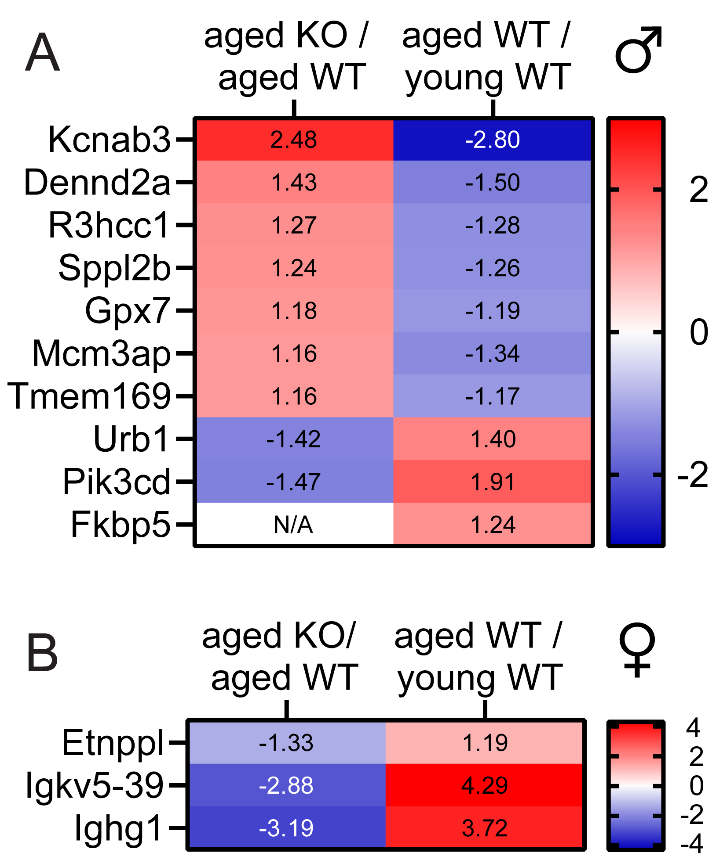


**Figure S6: List of proteins demonstrating altered expression patterns during aging yet displaying a reversed expression profile upon *Fkbp5* KO in the amygdala.** List of proteins in the amygdala of **A** males and **B** females that overlapped between the *Fkbp5* KO vs aged wild-type and aged wild-type vs young wild-type and corresponding z-score. Red colors represent upregulation and blue colors represent downregulation. (Welch’s t-test p < 0.05 and |z-score| >1).

**
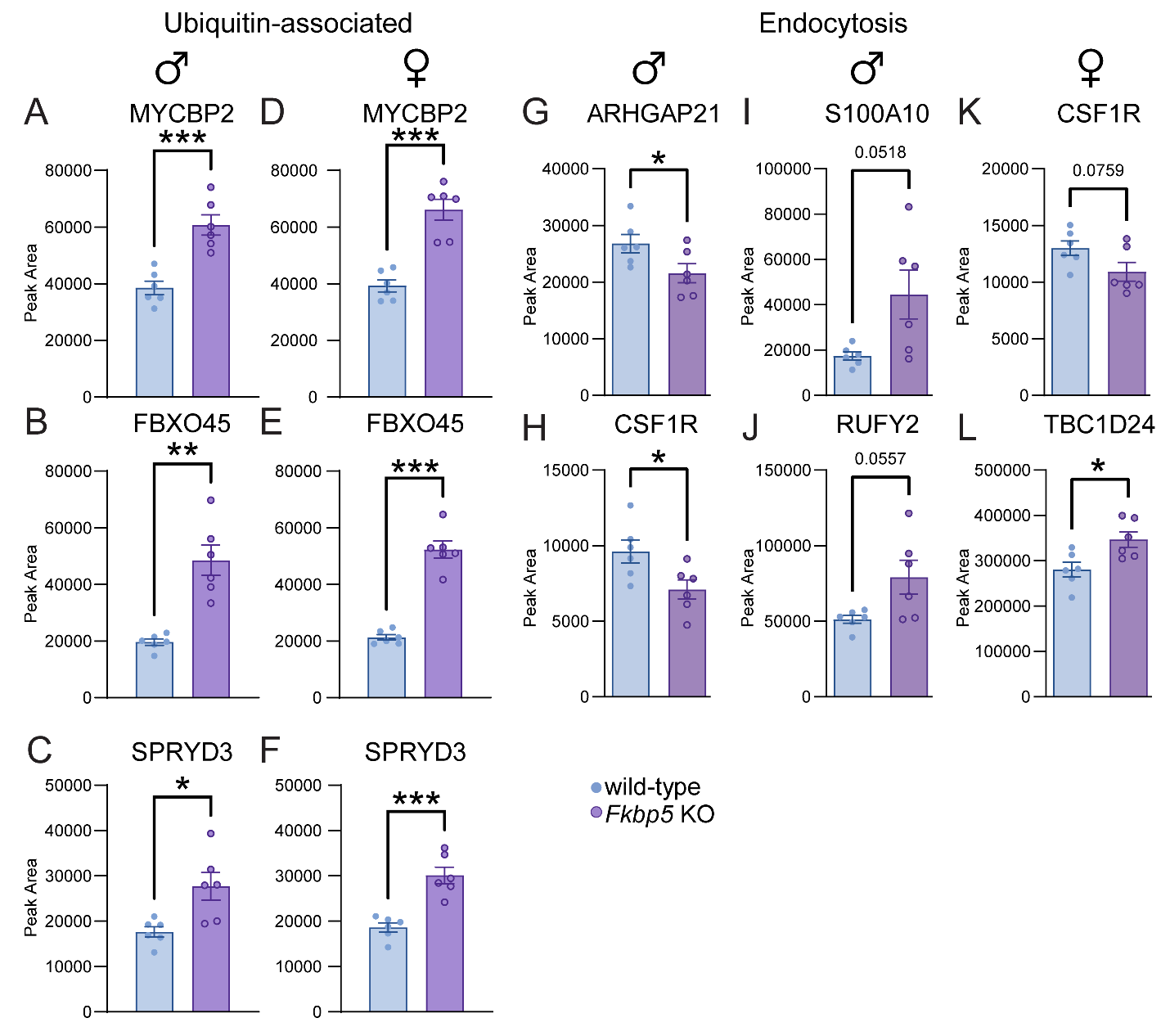
**

**Figure S7: Parallel reaction monitoring (PRM) of representative DEPs and associated pathways.** PRM analysis of proteins associated with **A-F** ubiquitination and **G-L** endocytosis in aged *Fkbp5* KO (n = 6 males and 6 females) and aged wild-type (n = 6 males and 6 females). Comparison done using unpaired t test. Statistical significance is indicated by *p < 0.05, **p < 0.01, ***p < 0.001.


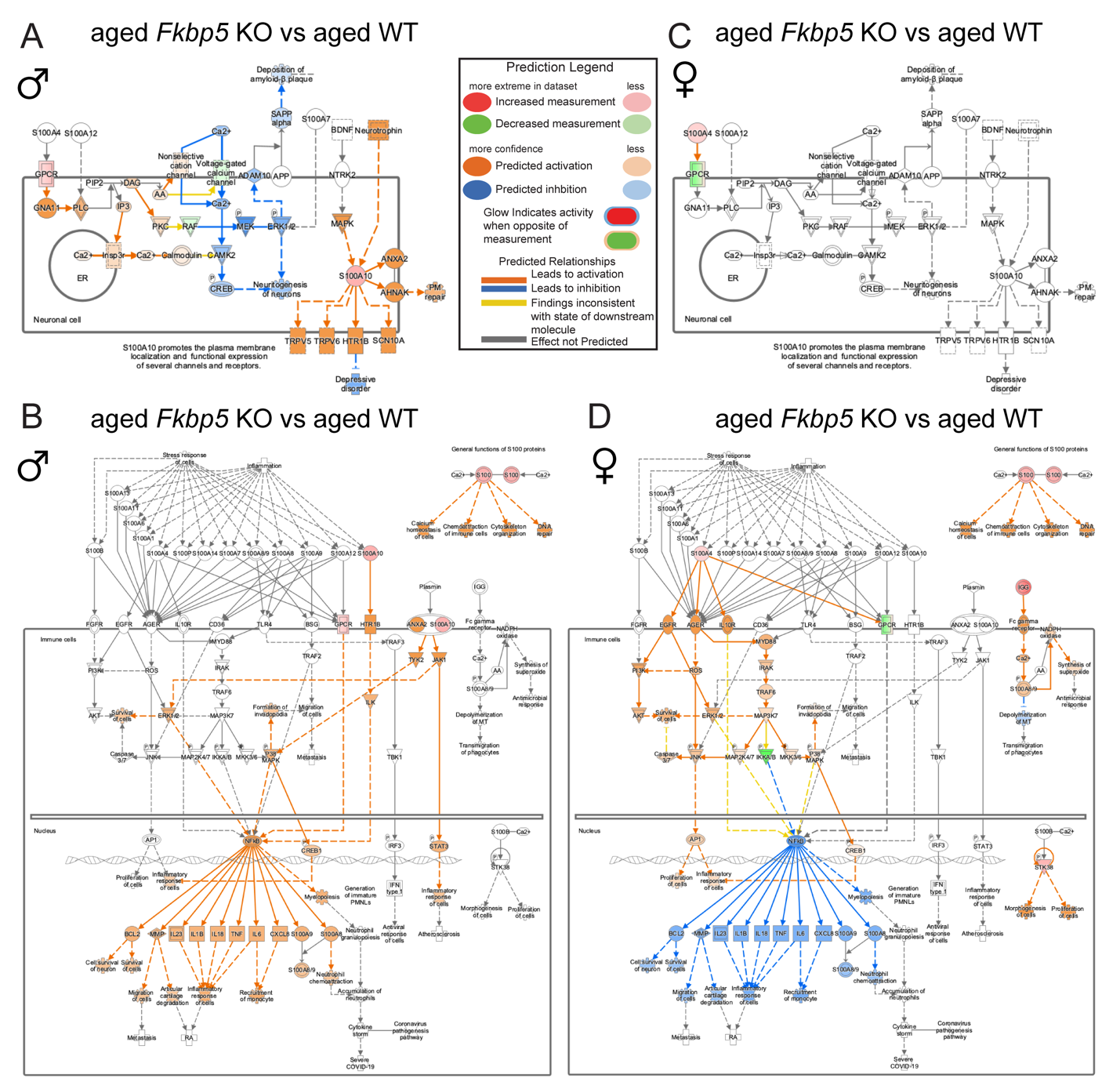


**Figure S8: Predicted activity of *S100* mediated NFκB signaling in *Fkbp5* KO hippocampus of male and female mice.** Ingenuity Pathway Analysis of *S100* signaling pathway in males in **A** neuronal and **B** immune cells as well as female **C** neuronal and **D** immune cells. Orange and blue colors indicate predicted activation and inhibition respectively while red and green indicate experimentally determined up and downregulated proteins that underlie the subsequent pathway activity predictions, respectively.


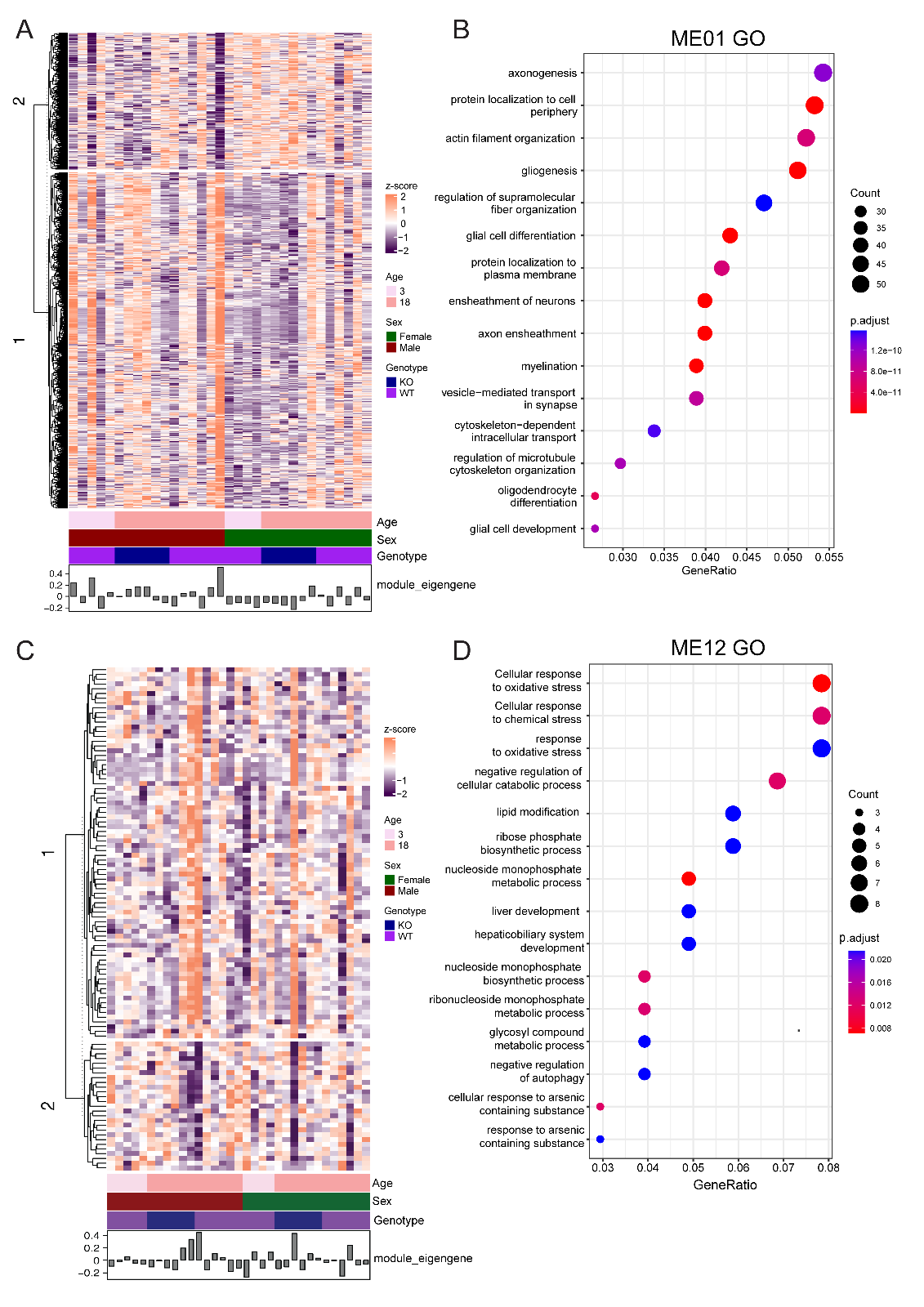


**Figure S9: Identification of gene modules and associated biological processes with correlations to specific traits. A** Heat map of protein expression profile and **B** Top 15 biological processes in module ME01. **C** Heat map of protein expression profile and **D** Top 15 biological processes in module ME12. Each row and column in the heatmaps correspond to a protein or a sample. The color from violet to orange means the z-score of a specific protein among all the samples in this study. The module eigengene is considered a representative of the gene expression profiles in a module. Dot plots represent pathways with the most significant adjusted p-value are plotted in order of protein ratio. The color of the dots represents the adjusted p-value of the GO term, and the size of the dots represents gene counts corresponding to the GO term.


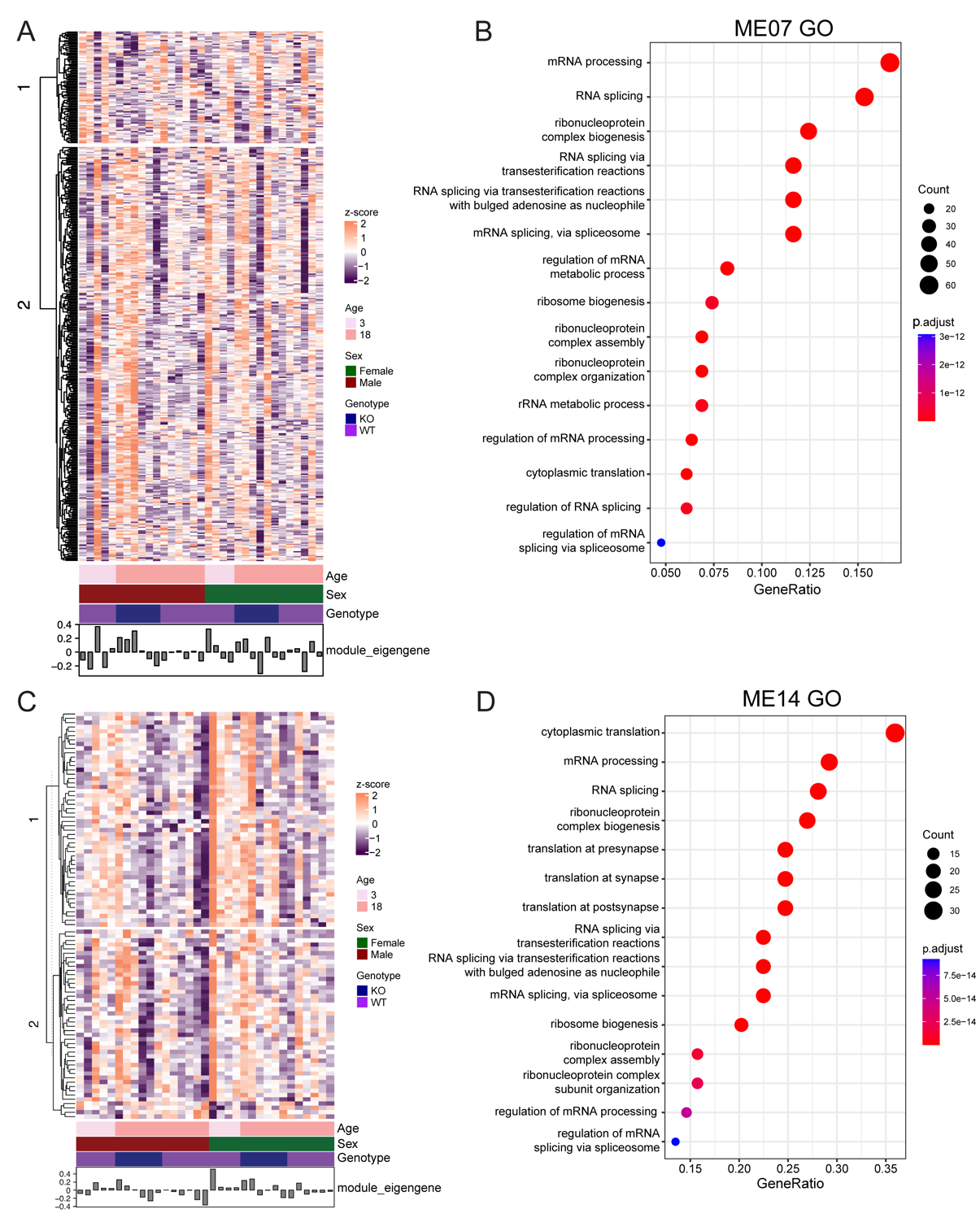


**Figure S10: Identification of gene modules and associated biological processes with correlations to specific traits. A** Heat map of protein expression profile and **B** Top 15 biological processes in module ME07. **C** Heat map of protein expression profile and **D** Top 15 biological processes in module ME14. Each row and column in the heatmaps correspond to a protein or a sample. The color from violet to orange means the z-score of a specific protein among all the samples in this study. The module eigengene is considered a representative of the gene expression profiles in a module. Dot plots represent pathways with the most significant adjusted p-value are plotted in order of protein ratio. The color of the dots represents the adjusted p-value of the GO term, and the size of the dots represents gene counts corresponding to the GO term.
